# Circ_0000235 targets MCT4 to promote glycolysis and progression of bladder cancer by sponging miR-330-5p

**DOI:** 10.1038/s41420-023-01582-z

**Published:** 2023-08-02

**Authors:** Jianye Zhong, Abai Xu, Peng Xu, Minhong Su, Peng Wang, Zhe Liu, Boping Li, Chunxiao Liu, Ning Jiang

**Affiliations:** 1 Department of Urology, Zhujiang Hospital, Southern Medical University, Guangzhou, China; 2 Laboratory of Urology, Zhujiang Hospital, Southern Medical University, Guangzhou, China; 3 Department of Respiratory and Critical Care Medicine, Zhujiang Hospital, Southern Medical University, Guangzhou, China

**Keywords:** Bladder cancer, Mechanisms of disease

## Abstract

Warburg effect plays a crucial role in bladder cancer (Bca) development. However, the mechanism by which glycolysis is involved in Bca remains poorly understood. CircRNAs commonly play a regulatory role in tumor progression. Our study discovered and identified a novel circRNA, *hsa_circ_0000235* (circ235), and investigated its role in the glycolytic process, which further results in the progression of Bca. We applied qRT-PCR to assess its clinicopathological relevance and evaluated its proliferation, migration, and glycolytic capacity. We investigated its mechanism using RNA immunoprecipitation, dual-luciferase reporters, and fluorescence in situ hybridization. The findings demonstrated that circ235 was dramatically increased in Bca tissues and was related to a worse prognosis. In vitro studies revealed that circ235 accelerated the rate of extracellular acidification and promoted glucose uptake and lactate manufacture in Bca cells. Additionally, it strengthened the proliferative and migratory capacities. Experiments on animals revealed that downregulating circ235 dramatically reduced carcinogenesis and tumor growth. Circ235 activates monocarboxylate transporter 4 (*MCT4*) by sponging *miR-330-5p*, which promotes glycolysis and tumor growth. In conclusion, these findings suggest that circ235 may be a viable molecular marker and therapeutic target for Bca.

## Introduction

Bladder cancer (Bca) is prevalent urothelial malignancy and ranks seventh among the most commonly diagnosed cancer in men globally [1]. About 524,000 Bca cases were diagnosed and 229,000 Bca-related deaths were recorded in 2019 [2]. The majority of Bca patients suffer from non-muscle invasive bladder cancer (NMIBC), while the remainder suffer from muscle invasive bladder cancer (MIBC) or metastatic Bca [3, 4]. Cisplatin-based combination therapy has been the primary treatment option for MIBC and metastatic Bca for approximately 20 years. However, its therapeutic effectiveness is limited [5]. In recent years, immunological agents targeting programmed cell death 1 (*PD-1*) and programmed cell death ligand 1 (*PD-L1*) pathways have emerged, providing a new therapeutic option [6]. However, the response rate for *PD-1*/*PD-L1* agents in Bca patients is approximately 20%, consequently, immunotherapy does not significantly advance the efficacy of Bca treatment compared to classical Bacillus Calmette-Guérin (BCG) therapy [7, 8], and may be partly due to an immunosuppressive tumor microenvironment caused by exacerbated glycolytic metabolism [9].

According to Otto Warburg, cancer cells reprogram their glucose metabolism to generate large quantities of lactate, even under aerobic conditions [10], to support growth, proliferation, and long-term survival [11]. This phenomenon was later known as the Warburg effect. Extracellular acidity increases the capacity of cancer cells for invasion and migration; thus, boosting the metastatic cascade. Moreover, lactate serves as a fuel for cancer cells, induces immunosuppression, and causes treatment refractoriness by activating hypoxia-inducible factor (*HIF1*) and the vascular endothelial growth factor (*VEGF*) signaling pathway [12, 13]. *HIF1* is a crucial mediator of chronic glucose uptake in Bca, as well as in other malignancies [14], and upregulation has been linked to cisplatin resistance and Bca aggressiveness [15].

Numerous members of the Solute Carrier (*SLC*) family, which includes at least 362 transporters, mutated and overexpressed in many diseases and cancers [16]. Monocarboxylate transporters (*MCT*s) encoded by *SLC16A* link the bidirectional movement of lactate, pyruvate, and other monocarboxylates penetrating plasmalemma [17]. Both the *MCT1* and *MCT4* isoforms, which are encoded by the *SLC16* gene, have attracted considerable attention by researchers. *MCT1* has a modest affinity for substrates and is created for both cellular uptake and efflux [18]. Low-affinity transporter *MCT4* selectively exports lactate and is mainly observed in regions with high glycolytic activity levels [18]. Because of their dual functions in lactate outflow and pH modulation, both proteins are essential for the glycolytic character in malignancies and have been linked to tumor aggressiveness and poor prognosis [19, 20]. Following *MCT4* knockdown in UC13, UC16, and T24 cells, lactate accumulation and cell growth were reduced, while reactive oxygen species (ROS) synthesis and apoptosis were promoted, and a decrease in tumor burden was observed in UC16 cell xenografts in mice [21]. Although evidence suggests that *MCT4* is implicated in the Warburg effect in Bca, the mechanisms through which it is regulated remain poorly understood.

Circular RNA (circRNA) is a category of non-coding RNA that is mainly generated through pre-mRNA back-splicing by combining a downstream 5' and an upstream 3' splice site [22]. As a result, the RNA molecule has a circular shape and a 30–50 phosphodiester binding at the area of the back-splicing junction [22]. CircRNAs have higher stability than linear RNA molecules because of their resistance to ribonuclease (RNase) breakdown [23–25]. CircRNAs are mainly located in the cell plasma, frequently contribute to indirectly controlling expression by sponging microRNAs (miRNAs) and reducing miRNA-mediated repression of downstream target genes [26–28]. Upon interactions with RNA-binding proteins (RBPs), circRNAs modulate a series of biological reactions by serving as protein scaffolds, miRNA sponges, and protein recruiters [29, 30]. Internal ribosome entry sites (IRES) are detected in some circRNAs and can induce cap-independent translation [31, 32]. In addition, researchers have demonstrated that circRNAs may also control glycolysis in cancer cells by modulating glucose metabolism and thus contributing to the development of malignant tumors [33–35]. Following *HIF1-α*-mediated activation of exosomal circPDK1, it activates the bromodomain PHD finger transcription factor (*BPTF*)/ cellular-myc (*c-myc*) axis by sponge adsorption of *miR-628-3p*. CircPDK1 also serves as a skeleton to strengthen the contact of ubiquitin-conjugating enzyme E2O (*UBE2O*) with bridging integrator 1 (*BIN1*), causing *UBE2O*-mediated degradation of *BIN1* and accelerating the advancement of pancreatic cancer [34]. By associating with *miR-338-3p*, circular methionine adenosyl transferase 2B (circMAT2B) regulates the expression of pyruvate kinase M2 (*PKM2*) to alter glycolysis and accelerates hepatocellular carcinoma progression [35]. However, whether circRNAs can affect tumor advancement by modulating glucose metabolism in Bca remains unclear.

Our previous study indicated that circular elongator acetyltransferase complex subunit 3 (circELP3) was elevated in hypoxic Bca, played a role in drug resistance and Bca development, as well as in the adaptive response to hypoxia [36]. As the Warburg effect dominates in the tumor microenvironment, in this study, we focus our attention on circRNA molecules that can regulate glycolysis and malignant progression of Bca, investigate their specific biological roles, and determine their potential as molecular markers and therapeutic targets for Bca.

## Results

### Validation of circ235 and its expression in Bca tissues and cell lines

**Fig. 1:**
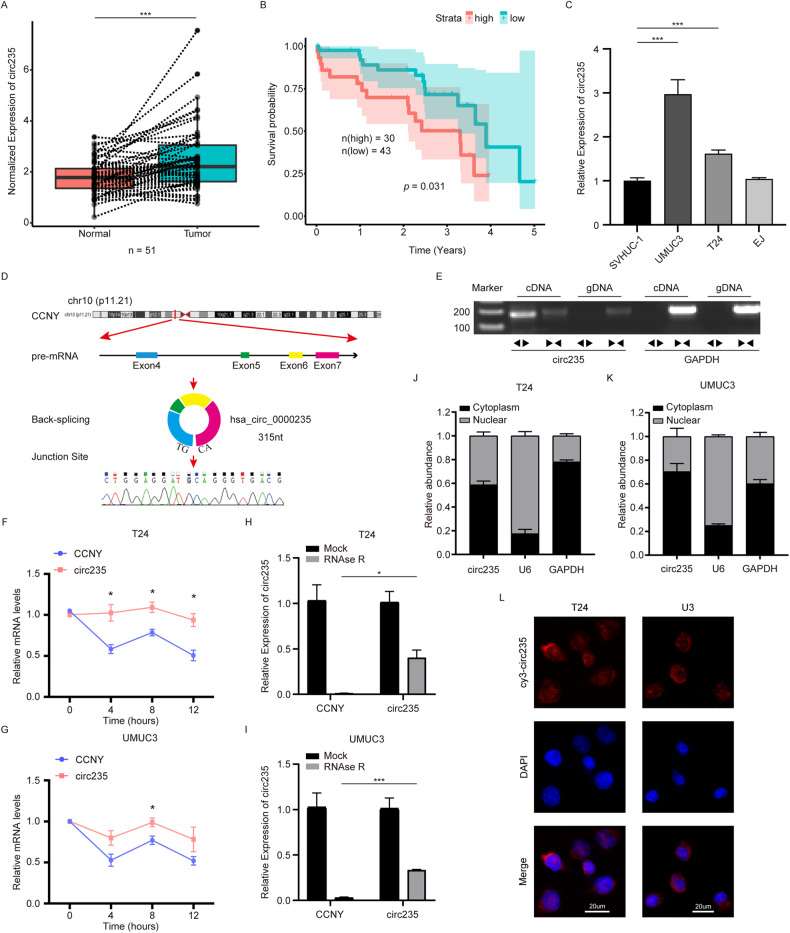
Validation and expression of circ235 in Bca tissues and cells. **A** Circ235 expression was observed to be considerably greater in Bca tissues than in adjacent normal tissues. (*n* = 51). **B** Correlation between circ235 levels (n_high_ = 30, n_low_ = 43) and overall survival rate for Bca patients. **C** Circ235 was highly expressed in multiple Bca cell lines in comparison to the control urinary epithelium SV-HUC-1 cell line. **D** A schematic diagram was constructed to illustrate the generation of circ235, which was derived from *CCNY* pre-mRNA through exon 7 ~ exon 4 back splicing (*hsa_circ_0000235*, 315nt), and the splice junction site was verified using Sanger sequencing. **E** RT-PCR and agarose gel electrophoresis were carried out in T24 cells to validate the existence of circ235. The control was *GAPDH*. **F**, **G** The relative abundance of circ235 and *CCNY* was analyzed using qRT-PCR in Bca cells exposed to actinomycin D at the specified time point. **H**, **I** The relative mRNA expression of circ235 and *CCNY* was analyzed using RT-qPCR following treatment with RNase R in Bca cells. **J**, **K** The relative abundance of circ235 in Bca cells’ cytoplasm and nucleus was determined by qRT-PCR, *U6* serving as a control for the nucleus, while *GAPDH* serving as a control for the cytoplasm. **L** Circ235 was predominantly sited Bca cells’ cytoplasm based on RNA FISH. Scale bar = 20 μm. *p < 0.05, **p < 0.01, ***p < 0.001, ****p < 0.0001.

To identify and study the role of circRNA in the glycolysis of Bca, we previously found that circELP3, which is elevated in a hypoxic environment, could regulate chemotherapy resistance in Bca cells and promote tumor progression [36]. While hypoxia can induce glycolysis, our further exploration and understanding of the bladder cancer microenvironment prompted us to pay more attention to aerobic glycolysis and its relationship with circular RNAs. As such, we have identified a novel and previously uncharacterized circRNA, *hsa_circ_0000235* (circ235). Circ235 was formed by reverse splicing four exons (exon 4 to 7) of the *CCNY* gene on chromosome 10. The whole length of circ235 was 315 nucleotides and Sanger sequencing was utilized to verify the junction site’s sequence (Fig. 1D). To prove its circular specificity, we used forward and reverse primers to amplify the genomic DNA (gDNA) and complementary DNA (cDNA) of the Bca cell line T24, and then performed agarose gel electrophoresis. We found that the reverse primer for circ235 could amplify bands with cDNA, but not gDNA, as a template (Fig. 1E). To clarify the expression of circ235 in Bca, we applied qRT-PCR to inspect expression in 51 cases of paired cancer and para-cancerous tissues in the Zhujiang Hospital (Guangzhou, China) and discovered that circ235 expression was considerably greater in malignant tissues compared to para-cancerous tissues. (*p* = 0.0001; Fig. 1A). Additionally, we detected the mRNA level of circ235 in the cancer tissues of 73 patients with Bca in our hospital and found that the higher circ235 expression was correlated with poorer patient prognosis (Fig. 1B). The patients’ clinical features are presented in Table 1. Next, we determined circ235 expression in the Bca cell lines and discovered that the abundance in T24 and UMUC3 was significantly higher than in the normal bladder epithelial cell line, SV-HUC-1. However, circ235 expression was only slightly elevated in the EJ cell line and was not considered statistically significant (Fig. 1C). As a result, T24 and UMUC3 cell lines were chosen for further investigations. To assess RNA stability in Bca cells, transcription inhibitor actinomycin D was used to treat Bca cells. In contrast to its linear host gene *CCNY*, which exhibited a progressive decline in mRNA expression over time, circ235 did not exhibit any apparent time-dependent decline in expression (Fig. 1F, G). Furthermore, we digested the Bca cell lines T24 and UMUC3 using RNase R. According to our findings, circ235 was partially digested; however, its stability remained much higher than that of *CCNY* mRNA (Fig. 1H, I). To understand the cellular localization of circ235, we performed nuclear plasma separation experiments in T24 and UMUC3 cells and noticed that circ235 was synthesized in both the cytoplasm and nucleus, with a greater level of expression observed in the cytoplasm (Fig. 1J, K). RNA FISH detections were also conducted on both Bca cell lines which further confirmed this phenomenon (Fig. 1L).

**Table 1:** The expression of circ235 and cancer features in Bca patients.

	No. of patients	circ235 expression		*p value*
		Low	High	
All patients	92	46	46	
Age (years)				0.2943
<65	41	23	18	
≥65	51	23	28	
Gender				0.3984
Male	86	44	42	
Female	6	2	4	
Tumor size				<0.0001
<2 cm	61	40	21	
≥2 cm	31	6	25	
Grade				0.1792
Low	17	11	6	
High	75	35	40	
Stage				0.2970
0–1	47	26	21	
2–4	45	20	25	
T Infiltrate (T)				0.4038
Ta–T1	48	26	22	
T2–T4	44	20	24	
Lymphatic metastasis (N)				0.9999
Yes	8	4	4	
No	84	42	42	
Distant metastasis (M)				0.3147
Yes	1	0	1	
No	91	46	45	

Chi-square test.

### Knockdown of circ235 inhibits the proliferation, migration, and glycolytic abilities of Bca cells

**Fig. 2:**
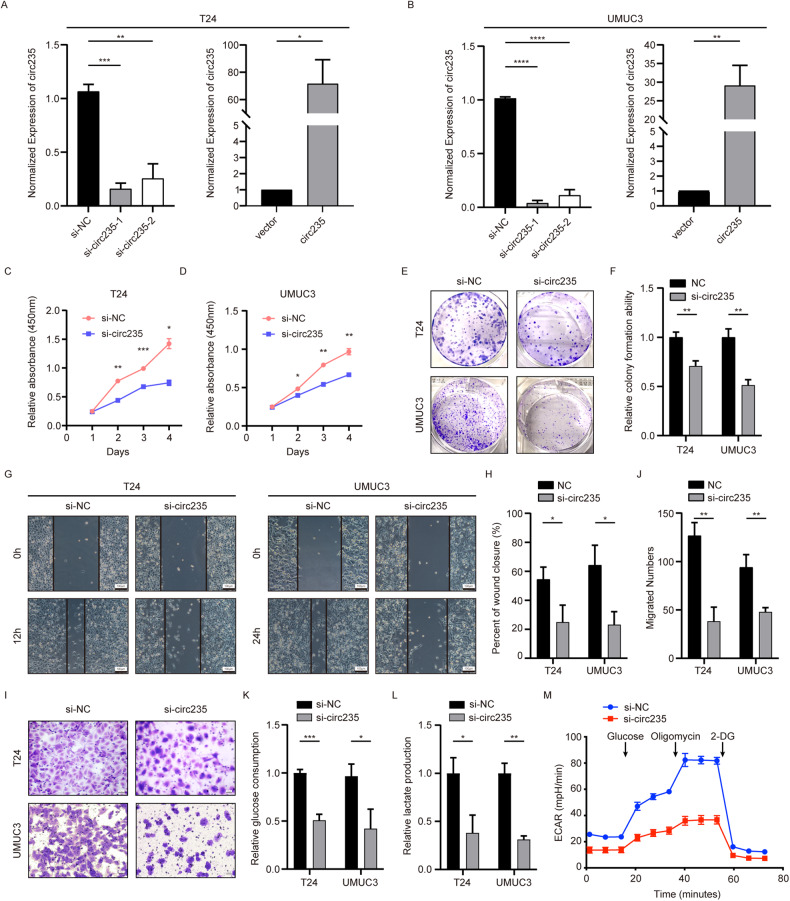
Circ235 promotes Bca cell migration, proliferation, and aerobic glycolysis. **A, B** T24 and UMUC3 cells underwent transfection with siRNAs targeting circ235 or were infected with circ235-overexpressing plasmids using lentiviral tools, and circ235’s relative expression was quantified using qRT-PCR and normalized using *β-actin*. **C**, **D** The significant reduction in Bca cells’ proliferative capacity was observed following knockdown of circ235. **E** Representative images of Bca cells underwent transfection with si-circ235 in the colony formation assay. **F** Statistical diagrams of Bca cells’ clonogenic abilities with circ235 knockdown. **G** Representative images of Bca cells underwent transfection with si-circ235 in the wound-healing assay. **H** Statistical diagrams of Bca cells’ migration abilities with circ235 knockdown in a wound-healing assay. **I** Representative images of Bca cells underwent transfection with si-circ235 in the transwell assay. **J** Statistical diagrams of T24 and UMUC3 cells’ migration abilities with circ235 knockdown in the transwell assay. **K** The relative glucose consumption of Bca cells with circ235 knockdown using the glucose assay kit. **L** The relative lactate production in Bca cells with circ235 knockdown using the lactate assay kit. **M** The extracellular acidification rate (ECAR) was quantitated using the Seahorse XFe96 Extracellular Flux Analyzer. *p < 0.05, **p < 0.01, ***p < 0.001, ****p < 0.0001.

To ascertain circ235’s impact on Bca cells’ in vitro ability, we established knockdown and overexpression systems in T24 and UMUC3 cells. Using small interfering RNAs (siRNAs) of two different sequences targeting the circ235 looping site, we found that si-circ235-1 had a higher knockdown efficiency in both cell lines (*p* = 0.0008; Fig. 2A, B); hence, this was defined as si-circ235 and used in all follow-up experiments. To facilitate subsequent rescue experiments, we used the plenti-ciR-copGFP-T2A-puro vector for lentiviral packaging to achieve overexpression of circ235 in Bca cell lines, and successfully established a stable overexpression system (Fig. 2A, B). First, we investigated the impact of circ235 on Bca cells’ proliferation and migration. The CCK-8 assay demonstrated that the proliferation rate of the si-circ235 group was considerably inferior to that of control group over time in both Bca cell lines (Fig. 2C, D). Additionally, the outcome of assay indicated that the clone formation ability was inferior to that of control group after knocking down circ235 (Fig. 2E, F). The wound closure experiment revealed that the ability of the circ235 knockdown group to heal scratches was impaired in both Bca cell lines (Fig. 2G, H). Analogous outcomes were observed in the transwell assay, where Bca cells were less able to migrate after circ235 expression was downregulated (Fig. 2I, J). To detect changes in metabolic capacity associated with hypoxia, such as glycolytic capacity, we tested glucose consumption and lactate production capacity in Bca cells and found that both were substantially decreased, compared to the control group (Fig. 2K, L). Furthermore, we performed a Seahorse assay to detect dynamic metabolic changes in T24 cells. After knocking down circ235, the extracellular acidification capacity was considerably reduced, which indicated impaired glycolytic capability (Fig. 2M).

### Suppression of circ235 inhibits tumor growth in vivo

**Fig. 3:**
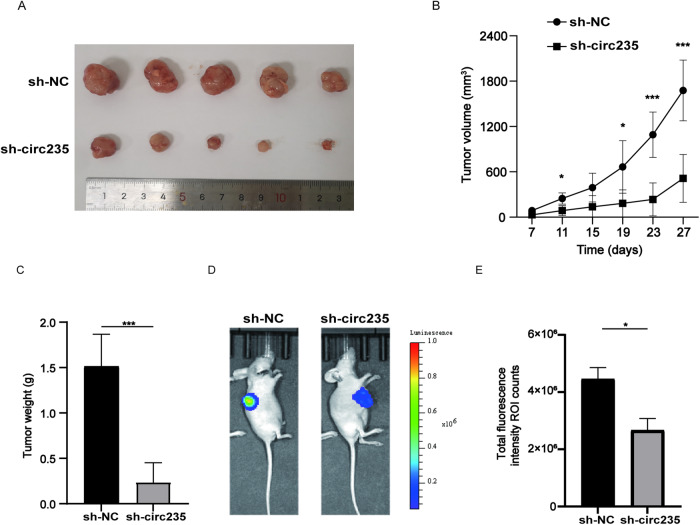
Circ235 promotes tumorigenesis in vivo. **A** 1 × 10^7^ T24 cells were injected hypodermically into mice, and typical photographs of tumors were obtained after 27 days. **B** Tumor volumes were recorded every 4 days from 7 days post-injection. **C** Tumor weights of mice in each group were recorded after dissection (*n* = 5 per group). **D**, **E** The progression of the subcutaneous tumor was tracked by an IVIS, and typical photographs of subcutaneous tumors were obtained. Total fluorescence intensity region of interest (ROI) counts was proceeded to detect the cellular activity of subcutaneous tumors (*n* = 3 per group). *p < 0.05, **p < 0.01, ***p < 0.001, ****p < 0.0001.

To determine whether circ235 accelerates tumor growth in vivo similar to our in vitro experiments, a tumorigenicity assay was performed using BALB/c nude mice hypodermically injected with infected T24 cells with sh-NC or sh-circ235 lentiviruses. After harvesting the subcutaneous tumors from mice on day 27 post-injection, we discovered that comparing to sh-NC group, the tumor weights and volumes in the circ235 knock-down group were much reduced (Fig. 3A, C). The sh-circ235 group also showed visibly slower tumor growth than sh-NC group did (Fig. 3B). The tumors were also imaged in vivo prior to harvesting, where it was revealed that comparing to sh-NC group, the tumor luminescence area and intensity in the sh-circ235 group were obviously greater (Fig. 3D, E).

### Circ235 serves as a sponge for miR-330-5p in Bca cells

**Fig. 4:**
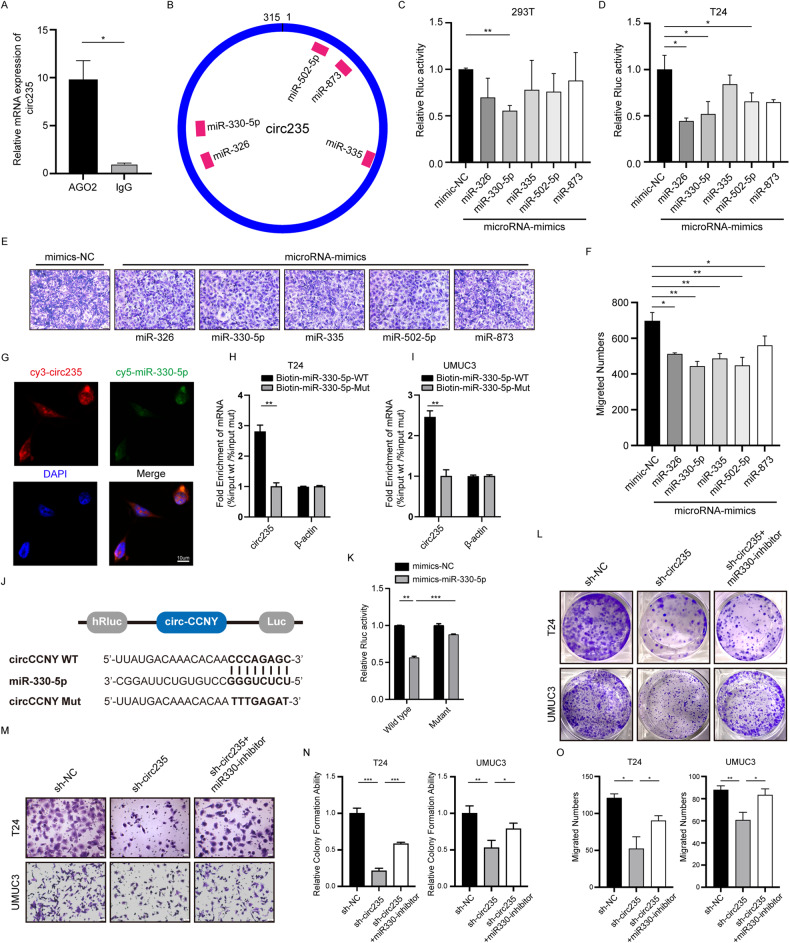
Circ235 functions as a sponge for miR-330-5p in Bca cells. **A** RIP was carried out to assess circ235 pulled down by anti-*AGO2* antibody or IgG in T24 cells. **B** Possible miRNAs binding to circ235 were predicted using CircInteractome, and top scores are listed in the diagram **C**, **D** The luciferase activity of 293T or T24 cells transfected with wild-type mimics, or controls, was investigated using a dual-luciferase reporter assay employing a psiCHECK-2 vector. The hRluc/fluc ratio was calculated to indicate luciferase activity. **E** Transfected T24 cells with the aforementioned mimics or controls were assessed for the capacity to migrate using a transwell assay. Scale bar = 25 μm. **F** Statistical diagram of the migratory capabilities of T24 cells that underwent transfection with the aforementioned mimics or controls in the transwell assay. **G** Circ235 and *miR-330-5p* were predominantly co-localized in Bca cells’ cytoplasm based on RNA FISH. Scale bar = 10 μm. **H**, **I** Biotin-labeled probes for wild-type or mutant *miR-330-5p* were applied to pull down RNA from Bca T24 or UMUC3 cell lines, and their relative input expression was detected by qRT-PCR with *β-actin* serving as the control. **J** The schematic illustrates the luciferase reporter vectors of circ-*CCNY* in both wild-type and mutant forms, revealing the presence of binding sites that are complementary to *miR-330-5p* within the circ-*CCNY* structure. **K** To determine the relative activity levels, luciferase activity intensity was measured in cells co-transfected with either *miR-330-5p* or NC mimics and the luciferase reporter vectors of circ235 wild or mutant type. Representative images (**L**) and statistical diagrams (**N**) of colony formation assays were implemented using three sets of T24 or UMUC3 cells (sh-NC, sh-circ235, and sh-circ235+*miR-330-5p* inhibitor). The addition of a *miR-330-5p* inhibitor partially rescued the proliferation of Bca cells infected with circ235-shRNA using lentiviral tools. Representative images (**M**) and statistical diagrams (**O**) of transwell tests were used to evaluate migratory capabilities of three sets of T24 and UMUC3 cells (sh-NC, sh-circ235, and sh-circ235+*miR-330-5p* inhibitor). The addition of a *miR-330-5p* inhibitor still partially rescued the migration of Bca cells infected with circ235-shRNA using lentiviral tools. *p < 0.05, **p < 0.01, ***p < 0.001, ****p < 0.0001.

Next, we explored the mechanism by which circRNAs function in Bca. CircRNAs that are predominantly expressed in the cytoplasm often exert their functions through competing endogenous RNAs (ceRNAs). Thus, we used RIP in the T24 cell line and found that intracellular RNA was pulled down by argonaute RISC catalytic component 2 (*AGO2*) protein or control immunoglobulin G (IgG). Using qRT-PCR, we observed that the *AGO2* protein group pulled down considerably more circ235 than the IgG control group (Fig. 4A), which suggested that circ235 may act through the functional adsorption of miRNAs. Thus, we employed the bioinformatics tool, Circinteractome, to predict potential microRNAs that could interact with *hsa_circ_0000235*. A total of 10 microRNA candidates were identified, including *hsa-miR-183*, *hsa-miR-326*, *hsa-miR-330-5p*, *hsa-miR335*, *hsa-miR-502-5p*, *hsa-miR-568*, *hsa-miR-583*, *hsa-miR-659* (refer to Supplementary Table S5). Filtering the candidates based on prediction scores, we retained those with scores of 90 or above, resulting in eight microRNAs showing higher binding potential. To ensure relevance to our study, we conducted an extensive literature review in the field of tumor research. Among the reviewed microRNAs, five (*miR-326*, *miR-330-5p*, *miR-335*, *miR-502-5p*, and *miR-873*) demonstrated consistency with our study. These five microRNAs were selected for further investigation in our study (Fig. 4B). Subsequently we constructed mimics of the five miRNAs mentioned above and *miR-330-5p* inhibitor, and have validated their efficiency by qRT-PCR (Fig. S1A). Obviously, the efficiency of the aforementioned mimics and *miR-330-5p* inhibitor are satisfactory; hence, they were used in follow-up experiments. We then performed the dual-luciferase reporter gene assay to examine the potential for circ235 to interact with these miRNAs, by embedding the circRNA sequence into a psiCHECK-2 dual luciferase plasmid vector. We found that the transfection of *miR-330-5p* mimics significantly decreased the luminescence intensity of this luciferase in 293T cells (*p* = 0.0035) (Fig. 4C), while *miR-326*, *miR-330-5p*, and *miR-502-5p* all significantly diminished the luminescence intensity in the Bca T24 cell line (*p* = 0.0158; Fig. 4D). Moreover, we carried out functional experimental screening on each of these predicted miRNAs to better select target miRNAs. As demonstrated in the transwell assay, all five miRNAs demonstrated significant reductions in migration ability compared to the control mimics, with *miR-330-5p* being the most pronounced (Fig. 4E, F). Several investigations have shown that *miR-330-5p* regularly has carcinoma suppressive role in Bca [37–39]. Thus, we selected *miR-330-5p* for all subsequent experiments. Using FISH in T24 cells, we found that Cy3-labeled circ235 and Cy5-labeled *miR-330-5p* were co-localized in Bca cells’ cytoplasm (Fig. 4G). To detect intracellular interactions, we designed and synthesized biotin-labeled probes targeting wild-type and mutant sequences of *miR-330-5p* to capture RNA from the Bca cell line. We found that the mutated *miR-330-5p* probe pulled less circ235 compared to the wild-type *miR-330-5p* probe, while the *β-actin* levels were comparable between the two groups (Fig. 4H, I). To explore the sites of *miR-330-5p* binding to circ235, we used bioinformatics tools to predict the possible binding sites and constructed psiCHECK-2 circ235 mutants (Fig. 4J). After transfecting negative controls (NC) and *miR-330-5p* mimics, dual luciferase reporter gene assays were executed. We observed that transfection of *miR-330-5p* in the wild-type vector significantly reduced the intensity of luciferase luminescence. However, *miR-330-5p* transfection in the vector with the mutated circ235 binding site had no significant impact on luciferase luminescence. Moreover, a significantly higher rate of luminescence intensity inhibition was observed in the wild-type group than in the mutant group (Fig. 4K). To clarify whether the possible binding of *miR-330-5p* to circ235 had any impact on the function of circ235, we performed functional rescue experiments. Cell colony formation and transwell assays were also used for validation, whereby we verified that the stable knockout of circ235 significantly reduced the clonogenicity of the cells in T24 and UMUC3, but the addition of *miR-330* inhibitor could partly rescue the effect (Fig. 4L, N). An identical design was applied to the transwell migration experiments, with similar results observed in both Bca cell lines. The stable downregulation of circ235 expression significantly inhibited Bca migration ability, but this was restored when *miR-330* inhibitor was administered (Fig. 4M, O).

### MCT4 serves as a direct target of miR-330-5p

**Fig. 5:**
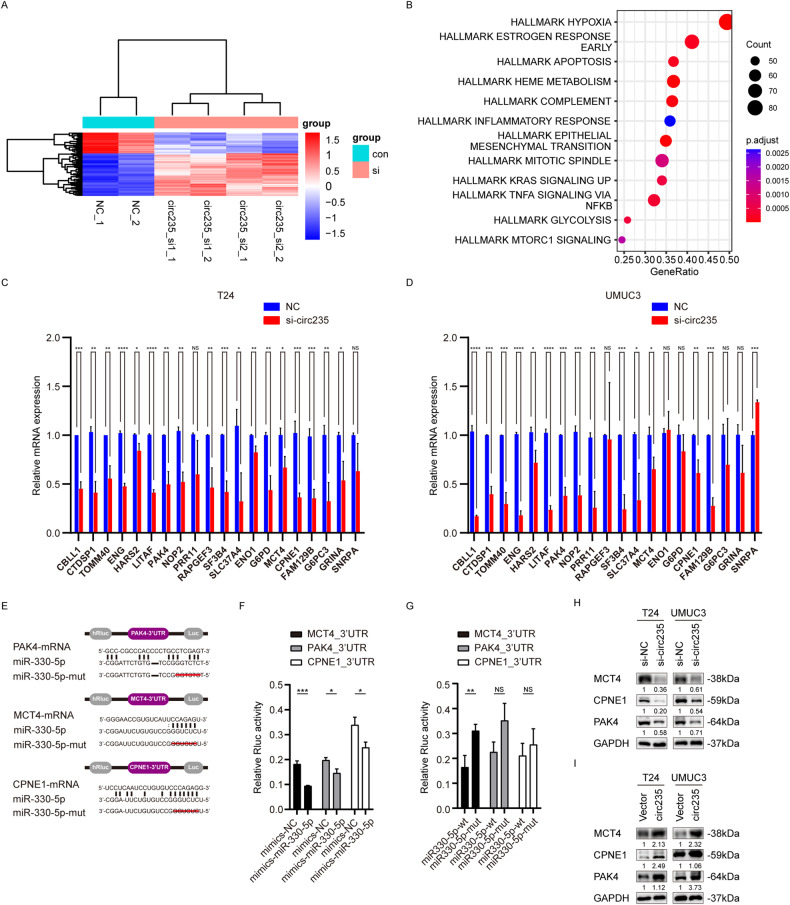
Circ235/miR-330-5p targets MCT4. **A** Following transfection of si-circ235-1, si-circ235-2, or the control, T24 cells were harvested, and high-throughput sequencing was employed to detect variably expressed mRNAs. The outcomes are visually demonstrated as a clustered heat map. **B** Dot plot of GSEA showing the significant differential gene set in Bca cells after circ235 knockdown. **C**, **D** The relative values of multiple predetermined genes’ expression were assessed in Bca cells that were transfected with either si-circ235 or control, utilizing qRT-PCR. **E** Schematic diagram of several complementary bonding regions between *miR-330-5p* and *PAK4*, *MCT4*, or *CPNE1*, including wild type and mutant. **F** In 293T cells co-transfected with either *miR-330-5p* mimics or NCs and luciferase reporter vectors integrated with the 3’UTR of *MCT4*, *PAK4*, or *CPNE1*, relative luciferase activities were measured. **G** In 293T cells co-transfected with wild-type or mutant *miR-330-5p* mimics and luciferase reporter vectors integrated with the 3’UTR of *MCT4*, *PAK4*, or *CPNE1*, measurements were conducted on relative luciferase activity intensity. **H** Western blot analysis of *PAK4*, *MCT4*, or *CPNE1* protein abundance in transfected Bca cells with si-circ235 or the control. **I** Western blot analysis assessed the *PAK4*, *MCT4*, or *CPNE1* protein levels in infected T24 or UMUC3 cells with circ235-overexpressing plasmids or the control using lentiviral tools. *p < 0.05, **p < 0.01, ***p < 0.001, ****p < 0.0001. NS not significant.

To determine which downstream genes were regulated following circ235 binding to *miR-330-5p*, we performed high-throughput sequencing of the mRNA expression profile in T24 cells. Two biological replicates were used to knock down siRNAs against two target sites of circ235 (Fig. 5A) and gene set enrichment analysis (GSEA) was then conducted. Hypoxic and glycolytic hallmarks were among the top gene sets in the differential analysis, which caught our attention since circ235 originated from previous hypoxia studies (Fig. 5B). Thus, we focused on the changes in these genes. We used qRT-PCR to validate the genes with the most significant changes in sequencing in Bca cells (Fig. 5C, D). Then, we selected the common genes that significantly decreased following circ235 interference for further bioinformatics analysis. The bioinformatics tools ENCORI and RNA22 were applied to forecast the target genes and binding sites of *miR-330-5p*. Among these, p21-activated kinase 4 (*PAK4*), *MCT4*, and copine 1 (*CPNE1*) were predicted to bind to *miR-330-5p* (Fig. 5E). Evidence suggests that *PAK4* kinase activity is amplified and overexpressed in Bca, associated with disease progression, and potential therapeutic target [40]. Similarly, *CPNE1* belongs to the copines family that is highly conserved and soluble in eukaryotes [41], and can augment aerobic glycolysis and facilitate the advancement of colon cancer by modulating the *AKT-GLUT1*/*HK2* pathway [42]. Given that *miR-330-5p* possesses a common sequence where each of these genes binds, we constructed deletion mutations of *miR-330-5p*. The 3’-untranslated region (UTR) sequences of the three genes were inserted into the psiCHECK-2 vector (Fig. 5E). Following *miR-330-5p* mimic transfection, a significant reduction in relative luciferase luminescence intensity was observed across all groups, in comparison to that in control group (Fig. 5F). Transfection of the mutant *miR-330-5p* resulted in an apparent decrease in the inhibitory effect on luciferase luminescence intensity in the *MCT4* group. The reduction was also observed in the *PAK4* and *CPNE1* groups, but the difference was not statistically significant (Fig. 5G). Using western blot analysis, we then detected the protein expression of *PAK4*, *MCT4*, and *CPNE1*. After knocking down the expression of circ235 using siRNA, these three genes’ values of protein expression were diminished in both Bca cell lines (Fig. 5H). Following lentiviral overexpression of circ235 in the Bca cell lines, *MCT4* and *PAK4* considerably increased in both T24 and UMUC3, while *CPNE1* only increased in the T24 cell line (Fig. 5I). Therefore, *MCT4* was selected as a downstream regulatory gene of interest for our functional studies.

### Circ235 modulates proliferation, migration, and glycolysis in Bca cells via miR-330-5p/MCT4

**Fig. 6:**
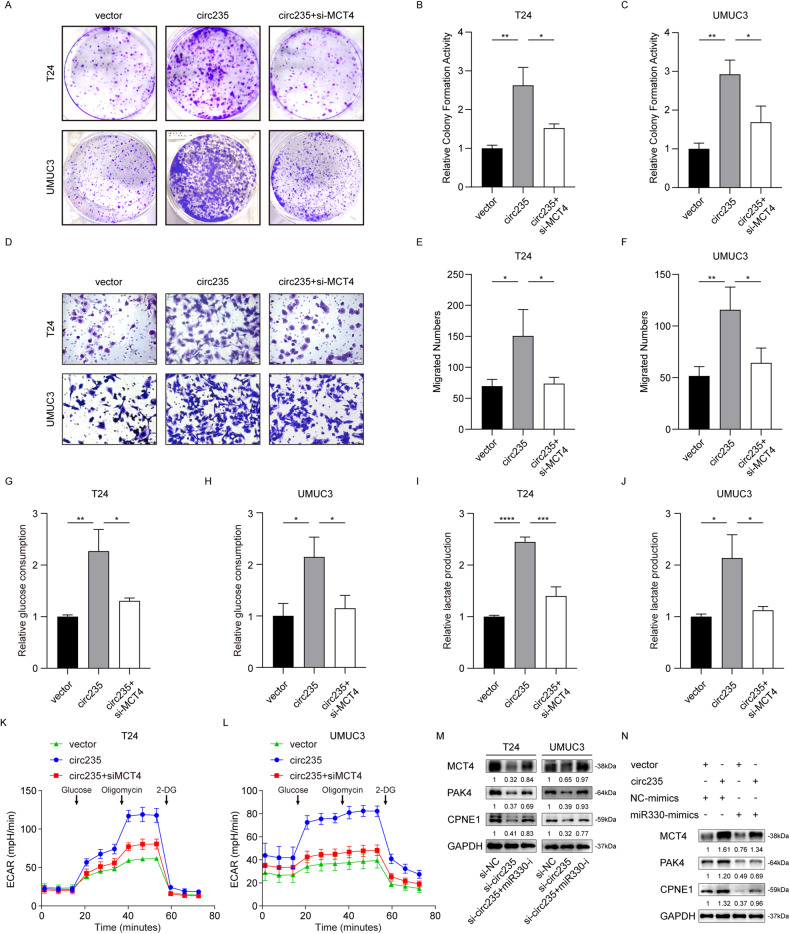
MCT4 promotes the migration, proliferation, and aerobic glycolysis of Bca cells via miR-330-5p. Exemplary photographs (**A**) and statistical diagrams (**B,** **C**) of colony formation assays were implemented using three sets of T24 or UMUC3 cells infected with circ235-overexpressing plasmids or the control using lentiviral tools, transfected with or without si-*MCT4* (vector, circ235, and circ235+si-*MCT4*). si-*MCT4* partially rescued the proliferative capacity of infected Bca cells with circ235-overexpressing plasmids using lentiviral tools. Exemplary photographs (**D**) and statistical diagrams (**E,** **F**) of transwell tests were used to evaluate migratory capabilities of the three sets of infected T24 and UMUC3 cells with circ235-overexpressing the plasmids or control using lentiviral tools, transfected with or without si-*MCT4* (vector, circ235, and circ235+si-*MCT4*). si-*MCT4* still partially rescued the migration of Bca cells infected with circ235-overexpressing plasmids using lentiviral tools. **G**, **H** Relative glucose consumption of three sets of Bca cells (vector, circ235, and circ235+si-*MCT4*) was calculated using the glucose assay kit. **I**, **J** Relative lactate production in three sets of Bca cells (vector, circ235, and circ235+si-*MCT4*) was detected using the lactate assay kit. **K**, **L** The ECAR of infected Bca cells with circ235-overexpressing plasmids or the control by lentiviral tools and transfected with or without si-*MCT4*, were quantified using a Seahorse XFe96 Extracellular Flux Analyzer. **M** Western blot analysis assessed *PAK4*, *MCT4*, or *CPNE1* protein levels in co-transfected T24 or UMUC3 cells with si-circ235 or the control and with or without *miR-330-5p* inhibitor. **N** Western blotting testing assessed the *PAK4*, *MCT4*, or *CPNE1* protein levels in T24 or UMUC3 cells that were infected with circ235-overexpressing plasmid or the control using lentiviral tools, while transfecting either *miR-330-5p* mimics or the control. *p < 0.05, **p < 0.01, ***p < 0.001, ****p < 0.0001.

To clarify the impact of *MCT4* on the downstream function of circ235, we performed functional rescue experiments. Before using siRNA targeting *MCT4*, we found that si-*MCT4* had a reliable knockdown efficiency in both cell lines (Fig. S1B); hence, this was used in follow-up experiments. The clonogenic ability was noticeably enhanced following lentiviral overexpression of circ235 in the Bca cells and this ability was partly recovered when *MCT4* was knocked down (Fig. 6A–C). Using a transwell assay, we observed increased cell migration ability after circ235 overexpression, which was also restored after *MCT4* knockdown (Fig. 6D–F). To verify the effect of *MCT4* on circ235 in Bca cell metabolism, we examined glucose consumption capacity (Fig. 6G, H) and lactate production capacity (Fig. 6I, J) in Bca cell lines. Circ235 overexpression had been demonstrated to dramatically increase glucose intake and lactate generation in T24 and UMUC3 cells, while knocking down *MCT4* significantly reversed glucose consumption and lactate generation capacity (Fig. 6G–J). To clarify this phenomenon, we conducted a real-time Seahorse assay using Bca cells and found that circ235 overexpression increased the ECAR, while lowering *MCT4* restored ECAR (Fig. 6K, L). Western blotting experiments were used to clarify whether *miR-330* affects downstream target genes’ product regulated by circ235. We discovered that the expression of *MCT4*, *PAK4*, and *CPNE1* decreased following the depletion of circ235, while the expression of these three genes was restored after the application of a *miR-330* inhibitor (Fig. 6M). Upon stable circ235 overexpression, the three target genes were induced and *miR-330* transfection partly restored expression (Fig. 6N). Accordingly, we conducted functional rescue studies to understand how *MCT4* affects *miR-330-5p*’s downstream function. Following suppression of *miR-330-5p* in the Bca cells, the clonogenic capacity was considerably strengthened, and this capacity was partially regained when *MCT4* was silenced (Fig. S2A–C). With a transwell experiment, we concluded that *miR-330-5p* repression raised cell migratory capacity, which was likewise recovered following *MCT4* silencing (Fig. S2D–F). Also we investigated the ability of Bca cell lines to consume glucose (Fig. S2G–H) and manufacture lactate (Fig. S2I–J) to confirm the impact of *MCT4* on *miR-330-5p* on the glucose metabolism of Bca cells. It has been observed that *miR-330-5p* inhibition considerably boosted glucose intake and lactate production in Bca cells, when *MCT4* knockdown significantly weakened both of these functions (Fig. S2G–J).

### Downregulation of MCT4 attenuates the carcinogenic effect of circ235 in vivo

**Fig. 7:**
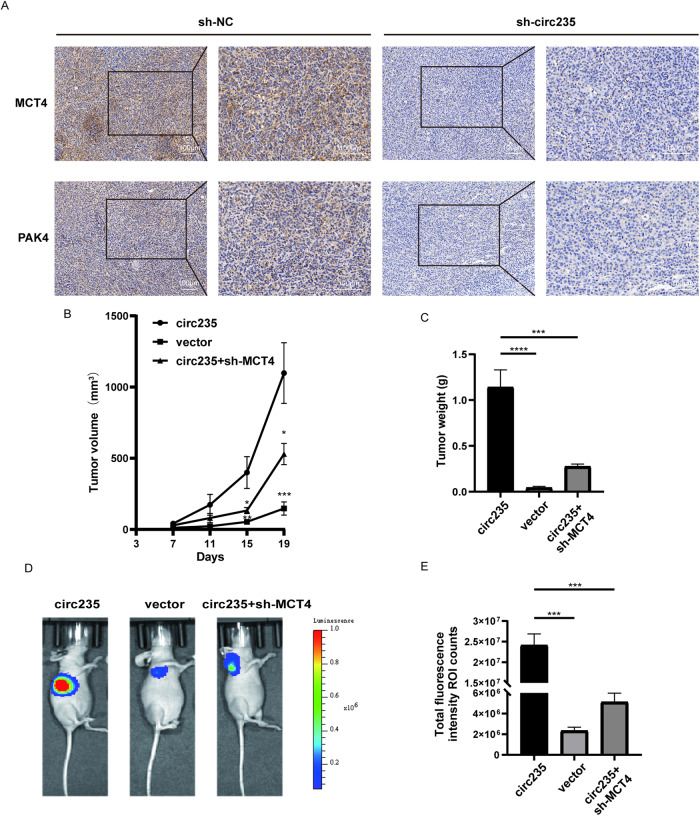
MCT4 regulates tumor progression in vivo. **A** *MCT4* and *PAK4* protein levels in the tumors were investigated using an immunohistochemistry assay. Scale bars = 100 μm. **B** Tumor volumes were recorded every 4 days from 7 days post-injection until the 19th day. **C** Tumor weights of mice in each group were recorded after dissection (*n* = 5 per group). **D**, **E** Typical IVIS photographs of three groups of subcutaneous tumors were obtained. Total fluorescence intensity ROI counts was proceeded to detect the cellular activity of subcutaneous tumors (*n* = 3 per group). *p < 0.05, **p < 0.01, ***p < 0.001, ****p < 0.0001.

Using immunohistochemical staining, we examined the expression of downstream regulatory genes in these tumors in mice. Our findings indicate that compared to the sh-NC group, the sh-circ235 group exhibited lower abundance of *MCT4* and *PAK4*. This suggests that circ235 and its target genes, *MCT4* and *PAK4*, may still play a role in mediating regulatory interactions in vivo in mice (Fig. 7A). In addition, we performed subcutaneous injections of nude mice using three sets of infected T24 cells: circ235-overexpressing plasmids, sh-*MCT4*, and control (vector, circ235, and circ235+sh-*MCT4*). After harvesting the subcutaneous tumors from mice 19 days post-injection, we observed a reversal in tumor volumes, weights, and growth in the circ235+sh-*MCT4* group compared to the overexpression group (Fig. 7B, C and Fig. S3). Furthermore, IVIS imaging of tumors in the circ235+sh-*MCT4* group showed similar reversion (Fig. 7D, E). Based on these findings, we have confirmed that *MCT4* is a downstream target in our study.

### Correlation of circ235/miR-330/MCT4 expression with the clinicopathological characteristics of Bca

**Fig. 8:**
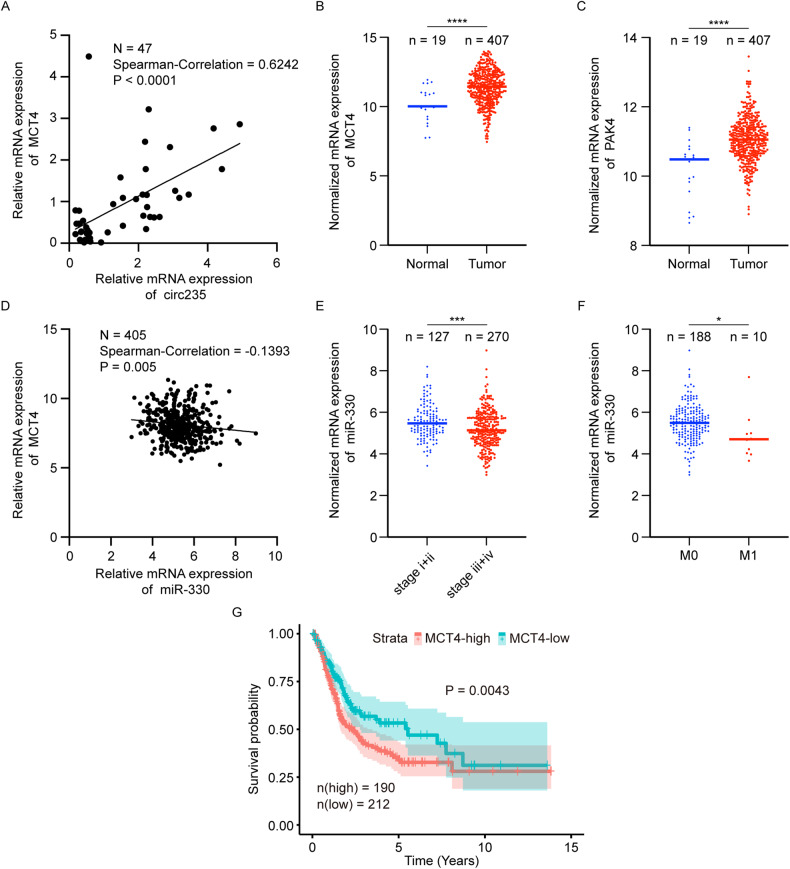
MCT4 and miR-330 validation and expression in Bca tissues. **A** Relevance between *MCT4* and circ235 expression in Bca tissues were collected from Zhujiang Hospital of Southern Medical University (*n* = 47, Spearman correlation = 0.6242, *p* < 0.0001). **B**–**G** All data were obtained from TCGA. **B**, **C** Differences in *MCT4* and *PAK4* expression between Bca tissues and adjacent normal tissues (n_normal_ = 19, n_tumor_ = 407). **D** Correlation between *MCT4* and *miR-330* expression in Bca tissues (*n* = 405, Spearman correlation = –0.1393, *p* = 0.005). The *miR-330* expression level was related to the patient characteristics of (**E**) clinical stage (n_stage i+ii_ = 127, n_stage iii+iv_ = 270) and (**F**) M stage (n_M0_ = 188, n_M1_ = 10). **G** Correlation between *MCT4* levels (n_high_ = 190, n_low_ = 212) and Bca patients’ overall survival rate. *p < 0.05, **p < 0.01, ***p < 0.001, ****p < 0.0001.

To determine whether circ235 and its related molecules have a clinical correlation, we studied patients from both our hospital and TCGA. First, the association between *MCT4* and circ235 expression in cancer tissue samples from 47 patients at our hospital was investigated using qRT-PCR. Using the Spearman method, we found that the correlation was positive, with a correlation coefficient of 0.6242 (*p* < 0.0001; Fig. 8A). Furthermore, *MCT4* and *PAK4* expression were markedly higher in cancerous tissues than in the paired para-cancerous tissues, according to an analysis of downstream gene expression conducted using Bca patient samples from TCGA (*p* < 0.0001; Fig. 8B, C). We performed a survival analysis of 402 patients using TCGA and found that overall survival was significantly lower in patients with high *MCT4* expression (*p* = 0.0043; Fig. 8G). The Spearman correlation of 405 patients from TCGA revealed a negative association between *miR-330* and *MCT4* expression, with a correlation coefficient of –0.1393 (*p* = 0.005; Fig. 8D). Further analysis of the relationship between *miR-330* and the clinical stage of Bca revealed that *miR-330* abundance notably decreased in the late stages (Fig. 8E). Following analysis of metastases, we discovered that M1 stage patients’ *miR-330* abundance in carcinoma tissues was less than that of M0 stage patients (Fig. 8F). Therefore, the correlation between circ235 expression and the clinical significance of downstream genes was supported using Bca patient data from our hospital and TCGA, which indicates a role for the regulatory circ235/*miR-330*/*MCT4* axis in glycolysis and Bca progression.

## Discussion

An increasing number of circRNAs have been discovered as a result of high-throughput sequencing [43]. CircRNAs have been demonstrated to be influential on numerous human malignancies, including Bca, hepatocellular carcinoma, and breast cancer [44–46]. circRNAs are novel gene regulators that regulate downstream factors, as well as tumor phenotypes, including migration, proliferation, and the Warburg effect [47], also considered as aerobic glycolysis, to generate abundant ATP to satisfy the requirements of the tumor microenvironment [11]. Generally, tumors driven by genomic instability frequently exhibit reprogramming of energy metabolism [48].

In this study, we revealed that circ235 promoted glucose uptake and lactate production in Bca cells. Kumagai et al. reported that lactate generated by tumor cells supports immune evasion by restricting the activity of effector T cells, which contributes to tumorigenesis [49]. Renner and Brand provided evidence that the high glycolytic state of tumors is correlated with resistance to *PD-1* blockade therapy [50, 51]. Furthermore, lactate generated by glycolysis in tumor cells reduced the anti-tumor effect of certain immune cells [52]. With the Seahorse Extracellular Flux Analyzer, we can access OCR and ECAR of living cells and get immediate results. Currently, most conventional techniques are intrusive, laborious, and only permit limited sample throughput. In comparison, the Seahorse analyzer sensor cartridges provide real-time, non-invasive monitoring of glycolysis and mitochondrial respiration, without the utilization of dyes or labels [53]. In a functional context, circular cullin 3 (circCUL3) significantly enhances ECAR in gastric carcinoma cells [54]. Interestingly, knockdown of circ235 also inhibited ECAR in Bca cells. In other words, circ235 may facilitate the Warburg effect in Bca and act as a metabolic regulator of tumor metabolism.

Hence, the next stage was to elucidate how circ235 functions in the advancement and carcinogenesis of Bca. First, we employed bioinformatic tools to predict the potential targets of circ235. Dual-luciferase reporter gene assays in both 293T and T24 cells demonstrated that *miR-326* had similar effects to *miR-330-5p*. Therefore, since the results obtained in the Bca cell line were analogous to those in 293T cells, our experimental results could be considered credible. One study showed that circular quiescin sulfhydryl oxidase 1 (circQSOX1) enhanced colorectal cancer (CRC) carcinogenesis by stimulating phosphoglycerate mutase 1 (*PGAM1*) expression via sponge adsorption of *miR-326* and *miR-330-5p*, which facilitated CRC’s immune evasion by triggering aerobic glycolysis [55]. Similarly, by sponge adsorption of *miR-326/330-5p* clusters, the proliferative capacity of nasopharyngeal carcinoma cells was enhanced in another study [56]. Thus, we can infer that *miR-330-5p*’s physiological functions are similar to *miR-326*’s. In the present study, we performed additional functional experiments and confirmed *miR-330-5p* as the vital target of circ235, which is comparable to previous study that demonstrated that the downregulation of *miR-330-5p* recovered Bca cells’ phenotype of malignant advancement through the inhibition of long non-coding RNA *HAGLROS*, which can accelerate cancer progression and metastasis [57]. Additionally, the reduced abundance of circFARSA hindered its capability to function in Bca cells and delayed malignant transformation by sponging *miR-330-5p* [37]. Overall, there is cumulative evidence to suggest that *miR-330-5p* suppresses glycolysis during carcinoma progression. Unsurprisingly, our findings were analogous findings, such that suppression of *miR-330-5p* expression partially reversed the negative modulation of tumor phenotype by silencing circ235.

Following GSEA and differential analysis of downstream modulators, hallmarks of hypoxia, glycolysis, and epithelial-mesenchymal transition (EMT) were prominent. Previous studies implicated *PAK4* in cancer-related energy signaling pathways, such as mediating oncogenic transformation [58]. In CRC, *CPNE1* has been shown to facilitate growth, mitochondrial respiration, and aerobic glycolysis via *AKT* signaling [42]. In ROS-induced aerobic glycolysis-preferring non-small cell lung cancer cell subtypes, the absence of *MCT4* blocked aerobic glycolysis and leads to apoptosis [59]. *PAK4, CPNE1*, and *MCT4* are predicted to each have *miR-330-5p* binding sites and are tightly linked to tumor metabolism or glycolysis. Meanwhile, there are currently no reports of their correlation with EMT in the context of EMT involvement in the development of tumors [60]; hence, EMT hallmarks were omitted in the present study. In addition, *PAK4* has rarely been reported to have glycolytic regulatory effects. Therefore, after several experiments, *MCT4* was ultimately selected as the target regulatory gene. *MCT4* is a proton-coupled transporter that is highly expressed in metastatic tumors at hypoxic or high glycolytic regions. In tumor cells, *MCT4*-mediated lactate secretion is crucial for malignancy development and maintaining the tumor microenvironment [61]. Previous Bca research also indicated that the downregulation of Jumonji Domain Containing 1 A (*JMJD1A*) expression suppresses glycolysis by down-regulating the abundance of genes engaged in glucose metabolism, including *MCT4* [62]. Currently, *MCT4* inhibitor development is in the preliminary stages, and almost all *MCT* inhibitors are unable of specifically binding *MCT4* [63]. In our research, we uncovered the feature of *MCT4* in the modulation of the glycolytic capacity and malignant phenotype of Bca cells. We found that MCT4 knockdown led to metabolic reprogramming and attenuation of these phenotypes via *hsa_circ_0000235* or *miR-330-5p*. Therefore, our study confirmed that circ235 and *miR-330-5p* can act as modulators of *MCT4*, establishing a new path for the regulation of *MCT4* in Bca.

In addition, although we confirmed that circ235 is involved in the glycolytic metabolism and development of Bca, however, circ235 is also expressed in a fractional proportion of the nucleus, and research into mechanisms including circRNA-binding proteins may lead to new findings. Taken together, the exact mechanism by which circ235 induces the progression of Bca and modifies its metabolic pattern deserves to be further explored.

In conclusion, this study revealed that circ235 was notably upregulated in Bca tissues relative to adjacent para-cancerous tissues. Circ235 with elevated abundance is strongly correlated with poorer patient prognosis. Through in vitro functional experiments, we clarified that circ235 facilitated the acceleration of extracellular acidification, augmented glucose uptake, and stimulated lactate production in Bca cells, indicating its role in metabolic reprogramming. Circ235 was also found to act as a ceRNA for *miR-330-5p*, thereby upregulating the *MCT4* abundance, which promoting glycolysis and tumor advancement in Bca. To summarize, our study characterized a novel circRNA, circ235 with a role in the regulation of cell proliferation, migration, and aerobic glycolysis via the *miR-330-5p*/*MCT4* axis in Bca. These findings imply that circ235 may be a promising molecular marker and therapeutic target for Bca (Fig. S4).

## Materials and methods

### Bca tissues collection

92 cases of Bca tissues and 51 cases of adjacent cancer tissues from Zhujiang Hospital, Southern Medical University were collected. Every patient has signed an informed consent document before surgical operation. The Medical Ethics Committee of Zhujiang Hospital approved the plan and procedure. Histopathological and clinical diagnoses were carried out by pathologists on tissue specimens, which were kept in an RNA stabilization solution called RNA later Soln. (Invitrogen, USA) and stored in a −80°C freezer for long-term storage.

### Cell lines

Human-derived Bca cell lines (T24, UMUC3, EJ) and human bladder epithelial cells (SV-HUC-1) were provided by ATCC (Manassas, USA). All cell lines were authenticated by the supplier using short tandem repeat analysis, they have been routinely monitored for their authenticity and mycoplasma contamination negativity. The UMUC3 and HEK-293T cells were incubated in a complete medium that contained DMEM basal medium, penicillin-streptomycin mixture, and fetal bovine serum in a ratio of 100:10:1. Similarly, RPMI-1640 complete medium was prepared by the same method for T24 and EJ cell cultures. All the aforementioned cells were grown in a constant 37 °C incubator with a CO_2_ concentration of 5%.

### Plasmid constructions and transfection

Short-hairpin RNA (shRNA) from Kidan Bioscience (Guangzhou, China) was applied to stably knockdown the target circ235. Moreover, the whole-length cDNA of circ235 was used to overexpress circ235 by cloning it into the vector plenti-ciR-copGFP-T2A-puro. Ige Biotechnology (Guangzhou, China) manufactured *miR-330-5p* wild-type and mutant mimics. siRNAs were assembled by Kidan Bioscience (Guangzhou, China). Pre-experimental sequencing confirms that all oligonucleotides are correct. Transient transfection experiments were performed using Lipofectamine 2000 (Invitrogen, USA). All the above-mentioned RNAs’ sequences are presented in Supplementary Table S1.

### RNA extraction and quantitative real-time polymerase chain reaction (qRT-PCR)

RNAiso Plus (TaKaRa, Japan) was employed to separate and harvest total RNA from Bca cell lines and clinical tissues. The Stratagene Mx3000P PCR instrument (Agilent Technologies, California, USA) was used to execute qRT-PCR experiments. Prime Script RT Master Mix (TaKaRa, Japan) and TB Green Premix Ex Taq II (Tli RNaseH Plus) (TaKaRa, Japan) were utilized for the analysis of circRNA and mRNAs. miRNA 1st Strand cDNA Synthesis Kit (Accurate Biology, China) was applied to the reverse transcription of miRNAs. All primers were obtained from Ige Biotech (Guangzhou, China) and Accurate Biology. The primer sequences are displayed in Supplementary Tables S2 and S3. The above experimental data were processed using *U6* as an internal reference for miRNAs and *β-actin* as an internal reference for circRNA and mRNAs.

### DNA agarose gel electrophoresis and Sanger sequencing

Pre-obtained cDNA and gDNA samples were added to the wells of 1% agarose gels, and the gels were placed in TAE buffer for electrophoresis to separate circ235 and linear CCNY mRNA. The internal standard control was *GADPH*. 50 minutes of 130 V electrophoresis were used. Subsequently, band intensity was observed using the DNA marker (Vazyme, Nanjing, China) under ultraviolet radiation conditions. The PCR amplicons were sequenced using the Sanger sequencing technology at ForeverGen Co., Ltd. (Guangzhou, China).

### Actinomycin D RNA stability assay

To assess RNA stability in Bca cells, actinomycin D (Act-D, 2 μg/ml, APExBIO, USA, Cat#A4448) was added to treat Bca cells according to the administration time of 4 h, 8 h, and 12 h, and the cells were harvested to extract RNA. qRT-PCR method was utilized to measure the relative quantities of circ235 and *CCNY* mRNA.

### RNase R digestion assay

2.0 μg of RNA was separated from T24 and UMUC3 cells and subjected to a 30 minutes digestion at 37 °C either with or without 4U RNase R (Epicentre, USA, Cat#RNR07250). qRT-PCR assay was conducted to examine the RNA abundance of circ235 and *CCNY*.

### Nuclear-plasma separation experiment

Bca cells’ nucleus and cytoplasm were separated by a PARIS Kit (Invitrogen, USA) based on the description in the instruction guide. Then, the mRNA levels of circ235, *GAPDH*, and *U6* in the nuclear and cytoplasmic components were examined by qRT-PCR.

### Data collection and bioinformatics analysis

Non-strand-specific transcriptome sequencing was conducted in three groups of our T24 cell samples with two biological replicates to screen downstream genes or pathways and the data have been uploaded to Gene Expression Omnibus (GEO) database (GSE233933). As for the public database, transcriptome data of mRNA, microRNA, and Bca’s relevant clinical data were from the cancer genome atlas (TCGA) [64]. Using the R software, transcriptome downstream analysis was carried out. The survminer package’s surv_cutpoint function was applied to calculate the optimal cutoff point for the survival analysis. The survival analysis plots were done with the ggsurvplot package. Paired expression analysis of patient samples was conducted by the ggplot2 package. The clusterProfiler package is assigned to achieve gene set enrichment analysis (GSEA) [65]. The processing code of R is available under request. MicroRNA binding sites of circ235 were predicted using the database CircInteractome [66]. Targets and bonding sites of *miR-330-5p* were forecasted via ENCORI [67] and RNA22 [68].

### Colony formation assay

On 6-well plates, treated cells were planted and nurtured for seven days in a constant 37 °C incubator at a concentration of 5% CO_2_. After cell colonies were washed twice with PBS buffer, they were fixed in paraformaldehyde solution at a concentration of 4% for 20 minutes, and the condition of the cells was assessed. Stained colonies were washed using a PBS buffer and subsequently, a high-definition camera was used to capture the condition of the colonies. Image J software (NIH, USA) was implemented to count the colonies.

### Cell counting Kit-8 (CCK-8) assay

Bca cells were washed and trypsinized, counted, and implanted into each well at a quantity of 1 × 10^3^ cells. 10 µl of CCK-8 (APExBIO, USA) was injected to every sample, following exposure for designated time. Absorbance values were recorded by microplate detector (BioTek Synergy H1, Agilent Technologies, USA) at 450 nm after incubation for 2 h.

### Wound-healing assay

The treated Bca cells were inoculated in 6-well plate and incubated for one day. Afterward, the cells were slowly and carefully scraped using a 10 μl pipette tip (timepoint 0 h) and the scratch site was rinsed with PBS three times, and representative images were photographed at designated timepoint. Migration ratio was determined by calculating the reduction in the distance across damaged area, and this value was normalized to the control value at 0 hours.

### Transwell migration assays

Transwell chambers (8 μm; Corning, USA) were used to accomplish the exploration on cell migration capacity. RMPI-1640 or DMEM medium without serum (100 μl) containing 8 × 10^4^ cells were transplanted into the upper chamber. In the lower compartment, 500 μl of DMEM or RMPI-1640 medium containing 10% FBS was injected. Afterward, the plates were transferred to the cell culture incubator and cultured until 24 h, then the cells were sequentially treated with fixation and staining. Using an inverted light microscope (Leica, Germany), migrating cells were measured and numbered in three random areas.

### Glycolysis analysis

The Glucose Assay Kit (Solarbio, China) was utilized to detect glucose consumption in Bca cells, while the Lactate Assay Kit (Solarbio, China) was employed to measure lactate production in these cells, following the provided instructions. Cells were washed and trypsinized, counted, and plated for seahorse analysis. Cells were seeded on an XFe96 cell culture microplate. Experiments were completed in XF assay specialized medium and analysis of the results was accomplished by a Seahorse Xfe96 Extracellular Flux Analyzer (Agilent Technologies, USA). Glucose (10 mM), oligomycin (1.0 M), and 2-Deoxy-D-glucose (2-DG, 50 mM) were administered, respectively, when indicated. Basal ECAR reports were generated by Wave Desktop software (Agilent Technologies, USA).

### Biotin-coupled miRNA capture

The biotinylated *miR-330-5p* pull-down probes were transfected into 2 × 10^6^ Bca cells for 24 hours. Afterward, the cells were harvested. Then, cells were sealed using streptavidin magnetic beads (Thermo Fisher Scientific, China) and placed at an environmental temperature of 4 °C for 2 hours, the mixture was gathered into reaction tubes to elute the biotin-labeled RNA complexes. A Rneasy Mini Kit (QIAGEN, China) was utilized to extract the bound RNAs from the magnetic beads. Circ235 expression abundance was quantified by qRT-PCR experiments.

### RNA immunoprecipitation (RIP) assay

The RNA Immunoprecipitation Kit (Geneseed, Guangzhou, China) was applied to execute the RIP testing. RNase and protease inhibitors were added to lysis buffer, which was prepared for lysis of Bca cells according to the kit instruction. RIP lysates were harvested and treated with magnetic beads conjugated with nonspecific IgG or anti-*AGO2* antibody (Proteintech, Wuhan, China, Cat#67934-1-Ig) in RIP buffer. The following day, the magnetic beads were rinsed, and the expression level of circ235 was quantified by qRT-PCR.

### Dual-luciferase reporter assay

The dual luciferase reporter plasmids were created and supplied by Ige Biotechnology using a psiCHECK-2 vector plasmid that contained both renilla luciferase (hRluc) and firefly luciferase (fluc) reporters. Then, the above-mentioned luciferase vectors were transfected together with *miR-330-5p* mimics (IgeBio) or NC-mimics by the aforementioned transfection reagent. Similarly, the aforementioned luciferase vectors were transfected together with wild-type or mutant *miR-330-5p* mimics (IgeBio) in 293T cells. Following a 48-hour transfection period, hRluc and fluc values were detected by a Dual-Luciferase Reporter System (promega, USA). Data were collected and the ratio of hRluc to fluc was computed and utilized to compare groups.

### RNA Fluorescence In Situ Hybridization (FISH)

Cy3-labeled circ235 probes and Cy5-labeled *miR-330-5p* probes were supplied from GenePharma (Shanghai, China). DAPI was applied to counterstain the cell nuclei. The FISH Kit (GenePharma, China) was employed for determining the signal strength of the probes based on the manufacturer’s recommendations state. AX confocal microscopy (ECLIPSE Ti2-E, Nikon, Japan) was utilized to capture the fluorescent images. These probes’ sequences are displayed in Supplementary Table S4.

### Western blot analysis

Cells were collected and disintegrated in RIPA lysis buffer (Tianya Biotech, China) containing protease inhibitors and obtain proteins. The BCA Protein Assay Kit (NCM Biotech, China) was applied to measure the protein samples’ concentration. 20 μg of protein samples was added sequentially to the SDS-polyacrylamide gels to proceed with electrophoresis and the subsequent transfer to the nitrocellulose (NC) membranes (Merck, Germany). NC membranes were incubated with specific primary antibodies overnight at the condition of 4 °C after being masked with 5% non-fat milk for 1 hour. The following day, NC membranes were treated with the secondary antibodies for an hour at ambient temperature after being three times rinsed with TBST solution. The protein signal strength was inspected by the ECL kit. The following antibodies appeared in this research: anti-*CPNE1* antibody (Cat#10126-2-AP); anti-*MCT4* antibody (Cat#22787-1-AP); anti-*ENO1* antibody (Cat#11204-1-AP); anti-*SLC37A4* antibody (Cat#20612-1-AP); anti-*PAK4* antibody (Cat#14685-1-AP); anti-*ALDOA* antibody (Cat#11217-1-AP); anti-*GAPDH* antibody (Cat#10494-1-AP), the aforementioned antibodies were all supplied by Proteintech (Wuhan, China); anti-*β*-*actin* antibody (Abways, China, Cat#AB0035).

### Xenograft tumor models

The Zhujiang Hospital’s Animal Care and Use Committee approved all animal research, and all experiments were conducted in compliance with their guidelines. Bestest Bio-Tech (Guangdong, China) supplied BALB/c immunodeficient mice (four-week-old female). Mice were randomly allocated to each group using the random number table method. Around 1 × 10^7^ stable T24 cells were hypodermically implanted into mice under anesthesia (five mice per group). One week after injection, vernier calipers were used to measure the lengths and widths of the tumor xenografts every four days, and calculate the volume by following this equation: volume = (width^2^ × length) × 0.5. Following 3–4 weeks, mice’s subcutaneous xenograft tumors were harvested, and tumors’ weight was quantified using a high-accuracy balance. The in vivo imaging system (IVIS Spectrum, PerkinElmer, USA) was utilized to track the advancement of the subcutaneous tumors.

### Statistical analysis

At least three biological repetitions of each experiment were accomplished. Blinding was not applied. Pre-specified effect sizes were not considered when determining the sample size. GraphPad Prism 7 (GraphPad Software, USA) was applied to conduct the statistical analyses. Unpaired Student t-test (two groups) or one-way ANOVA (more than two groups) was served to calculate statistical *p* values. The difference was statistically significant when the *p* value was set to under 0.05. The data were presented as Mean ± SD (standard deviation). *p < 0.05, **p < 0.01, ***p < 0.001, ****p < 0.0001. ns, not significant.

## Supplementary information

Supplementary Tables and Figures

Original Data File

Supplementary Figure Legends

## Data Availability

The current manuscript and the supplemental files contain all of the research data, and the corresponding authors can be contacted for any additional enquiries.
