# Supplementary material for: Circ_0000235 targets MCT4 to promote glycolysis and progression of bladder cancer by sponging miR-330-5p
Source: Cell Death Discov. 2023 Aug 2;9:283. doi: 10.1038/s41420-023-01582-z (PMC10397263; doi:10.1038/s41420-023-01582-z)
Supplement: Supplementary file 1 — Supplementary Tables and Figures [file 41420_2023_1582_MOESM1_ESM.pdf]

Table S1 SiRNA, miRNA mimics, and inhibitors sequences used in this research.

**SiRNA, mimics and inhibitors**	**Sequences**
siRNA control	TTCTCCGAACGTGTCACGT
hsa_circ_0000235 siRNA-1	CGUCACCCUGCAUCCUCCATT
hsa_circ_0000235 siRNA-2	CAUCGUCACCCUGCAUCCUTT
MCT4 siRNA	TCCTGGGCTTCATTGACATCT
mimics-NC	UCACAACCUCCUAGAAAGAGUAGA
hsa-miR-326 mimics	Sense(5'-3') CCUCUGGGCCCUUCCUCCAG<br>Antisense(5'-3') CUGGAGGAAGGGCCCAGAGG
hsa-miR-330-5p mimics	Sense(5'-3') UCUCUGGGCCUGUGUCUUAGGC<br>Antisense(5'-3') GCCUAAGACACAGGCCCAGAGA
hsa-miR-335 mimics	Sense(5'-3') UCAAGAGCAAUAACGAAAAAUGU<br>Antisense(5'-3') ACAUUUUUCGUUAUUGCUCUUGA
hsa-miR-502-5p mimics	Sense(5'-3') AUCCUUGCUAUCUGGGUGCUA<br>Antisense(5'-3') UAGCACCCAGAUAGCAAGGAU
hsa-miR-873 mimics	Sense(5'-3') GCAGGAACUUGUGAGUCUCCU<br>Antisense(5'-3') AGGAGACUCACAAGUUCCUGC
hsa-miR-330-5p inhibitor	GCCUAAGACACAGGCCCAGAGA

Table S2 Primers for qRT-PCR.

**Gene Symbol**	**Forward Primer**	**Reverse Primer**
hsa_circ_0000235	TCGAAATCCGAAGTGCCACC	GGAGGATGCAGGGTGACG
MCT4	ACTCCGTCTACCTCTTCAGC	ATGGCACTGGAGAACTTGTG
GAPDH	GGAGCGAGATCCCTCCAAAAT	GGCTGTTGTCATACTTCTCATGG
CBLL1	TGTGCAGCGAATTGAGCAGT	GCACGGGTAACAGGTTTTCCA
CTDSP1	AGACCCCAGTCCAATACCTG	GACGCTTCAACACGTAGACCT
TOMM40	TCGGGGAGTCCAACTACCAC	TGAGCGTTGAGACTGCCAC
ENG	GGCCAGCATTGTCTCACTTC	CGGCACACTTTGTCTGGATC
HARS2	TCCCTCCGCTATGACCTTACT	GCCACACCTTTCCAACATGAT
LITAF	ATGTCGGTTCCAGGACCTTAC	TACGAAGGAGGATTCATGCCC
PAK4	GGACATCAAGAGCGACTCGAT	CGACCAGCGACTTCCTTCG
NOP2	CCCAGTCCCTATTTAATGCTCCT	CGTGGTTCACCATACCATCTTCT
PRR11	AAAGATGGACCCATGCAGATAAC	TGCTTTCGGCGATGGTATAAG
RAPGEF3	GACCGGAAGTACCACCTTAGG	AGATTCCCACAACTTGGCTCC
SF3B4	GGAAGATGCTGACTATGCCATT	TGTTGTGAGCTGATGCTTTGTT
SLC37A4	CTCCACAGTACCTGTCTTTGC	ACCCACAGGTAAGGGGACAG
ENO1	GCCGTGAACGAGAAGTCCTG	ACGCCTGAAGAGACTCGGT
G6PD	AACATCGCCTGCGTTATCCTC	ACGTCCCGGATGATCCCAA
CPNE1	CACTGCGTGACCTTGGTTCA	CTCCCACATCCTGTAAAAGGAC
FAM129B	CTGACGGAGTTCCTCCAGTTC	GAGGTTCCCCGAGAAGACGAT
G6PC3	CGAGGCGCTACAGAACCAG	CACTCGGTGATGAGGCTGAT
GRINA	CTCACCCCAGCATGGAAACTA	CAAGGTCAGCACTAGGAACAC
SNRPA	CTTTCTGAGAATCCACCGAATCA	CCAGACGGACCTCCTTGAAG
β-actin	AATCTGGCACCACACCTTCTAC	ATAGCACAGCCTGGATAGCAAC
CCNY	TAGCACAGTCAGTCAACCAAAC	TGGTGGCACTTCGGATTTCG
U6	CGCTTCGGCAGCACATATACTA	TCACGAATTTGCGTGTCATCCT
ALDOA	ATGCCCTACCAATATCCAGCA	GCTCCCAGTGGACTCATCTG

Table S3 Specific upstream primers for miRNAs used in qPCR assays.

**miRNA**	**Sequences**
hsa-miR-326	CTCTGGGCCCTTCCTCCAG
hsa-miR-330-5p	TCTCTGGGCCTGTGTCTTAG
hsa-miR-335	TCAAGAGCAATAACGAAAAATGT
hsa-miR-502-5p	ATCCTTGCTATCTGGGTGCTA
hsa-miR-873	GTGCAGGAACTTGTGAGTCT

Table S4 Sequences of the FISH and pulldown probes used in this study.

**Gene name**	**Probe (5'-3')**
hsa_circ_0000235	5'CY3 - TTTGTCCTGGAGGATGCAGGGTGACGATGGC
hsa-miR-330-5p	5'CY5 - GCCTAAGACACAGGCCCAGAGA
hsa-miR-330-5p pulldown	UCUCUGGGCCUGUGUCUUAGGC - 3'Biotin
hsa-miR-330-5p mut pulldown	UGCCUGUGUCUUAGGC - 3'Biotin

Table S5 Predicted miRNA-circ235 interaction by Circinteractome.

**Circ-MiRNA Prediction**											
Circ-Mirbase ID	CircRNA (Top) - miRNA (Bottom) Pairing	Site Type	CircRNA Start	CircRNA End	3' pairing	local AU	position	TA	SPS	context+ score	context+ score percentile
hsa_circ_0000235 (5' ... 3')	AGCUGACGGCUGAAUGUGCCAUC										
									7mer-m8	299	305	0.003	0.019	-0.06	-0.007	-0.036	-0.201	94
**hsa-miR-183 (3' ... 5')**	UCACUUAAGAUGGUCACGGUAU										
hsa_circ_0000235 (5' ... 3')	UUAUGACAAACACAACCCAGAGC										
									7mer-m8	231	237	0.012	-0.027	-0.051	0.026	-0.066	-0.226	94
**hsa-miR-326 (3' ... 5')**	GACCUCCUUCCCGGGUCUCC										
hsa_circ_0000235 (5' ... 3')	UUAUGACAAACACAA-CCCAGAGC										
														7mer-m8	231	237	-0.035	-0.027	-0.051	0.026	-0.066	-0.273	96
**hsa-miR-330-5p (3' ... 5')**	CGGAUUCUGUGUCCGGGUCUCU										
hsa_circ_0000235 (5' ... 3')	ACAAUUAAAUGUGUCGCUCUUGC										
									7mer-m8	106	112	0.003	-0.032	-0.047	0.005	-0.018	-0.209	96
**hsa-miR-335 (3' ... 5')**	UGUAAAAAGCAAUAACGAGAACU										
hsa_circ_0000235 (5' ... 3')	CCUCCAGGACAAAUAGCAAGGAA										
									8mer-1a	19	26	0.003	-0.054	-0.109	0.01	-0.063	-0.46	99
**hsa-miR-502-5p (3' ... 5')**	AUCGUGGGUCUAUCGUUCCUA										
hsa_circ_0000235 (5' ... 3')	CAACCAAACCUCAAGUAUACAAU										
								7mer-1a	88	94	0.008	-0.023	-0.04	0.004	0.059	-0.066	92
**hsa-miR-568 (3' ... 5')**	CACACAUAUGUAAAUAUGUA										
hsa_circ_0000235 (5' ... 3')	GAUGAAAAUCUUCACCCUCUUUC										
									7mer-m8	187	193	0.012	0.015	-0.044	0.022	0.021	-0.094	93

<table>
<tr><td>hsa-miR-583 (3' ... 5')</td><td>CAUUACCCUGGAAGGAGAAAC</td><td></td><td></td><td></td><td></td><td></td><td></td><td></td><td></td><td></td><td></td></tr>
<tr><td>hsa_circ_0000235 (5' ... 3')</td><td>AUAGCACAGUCAGUCAACCAAAC</td><td rowspan="3">7mer-1a</td><td rowspan="3">74</td><td rowspan="3">80</td><td rowspan="3">0.004</td><td rowspan="3">0.01</td><td rowspan="3">-0.042</td><td rowspan="3">0.008</td><td rowspan="3">0.027</td><td rowspan="3">-0.067</td><td rowspan="3">85</td></tr>
<tr><td></td><td>| | | | | |</td></tr>
<tr><td>hsa-miR-659 (3' ... 5')</td><td>ACCCCUGGGAGGGACUUGGUUC</td></tr>
<tr><td>hsa_circ_0000235 (5' ... 3')</td><td>GACAAACACAACCCAGAGCAGAA</td><td rowspan="3">7mer-1a</td><td rowspan="3">235</td><td rowspan="3">241</td><td rowspan="3">0.001</td><td rowspan="3">0.026</td><td rowspan="3">-0.042</td><td rowspan="3">0.011</td><td rowspan="3">-0.052</td><td rowspan="3">-0.13</td><td rowspan="3">82</td></tr>
<tr><td></td><td>| | | | | |</td></tr>
<tr><td>hsa-miR-767-3p (3' ... 5')</td><td>UCUUUGGUACCCCAUACUCGUCU</td></tr>
<tr><td>hsa_circ_0000235 (5' ... 3')</td><td>UAGCAAGGAAAUACAGUUCCUGC</td><td rowspan="3">7mer-m8</td><td rowspan="3">32</td><td rowspan="3">38</td><td rowspan="3">0.012</td><td rowspan="3">-0.021</td><td rowspan="3">-0.057</td><td rowspan="3">0.02</td><td rowspan="3">-0.018</td><td rowspan="3">-0.184</td><td rowspan="3">95</td></tr>
<tr><td></td><td>| | | | | | |</td></tr>
<tr><td>hsa-miR-873 (3' ... 5')</td><td>UCCUCUGAGUGUUCAAGGACG</td></tr>
</table>

MiRNAs with potential interactions with has_circ_0000235 were predicted using the Circinteractome tool. Only miRNAs with scores higher than 90 were considered for further confirmation in the subsequent step.

A

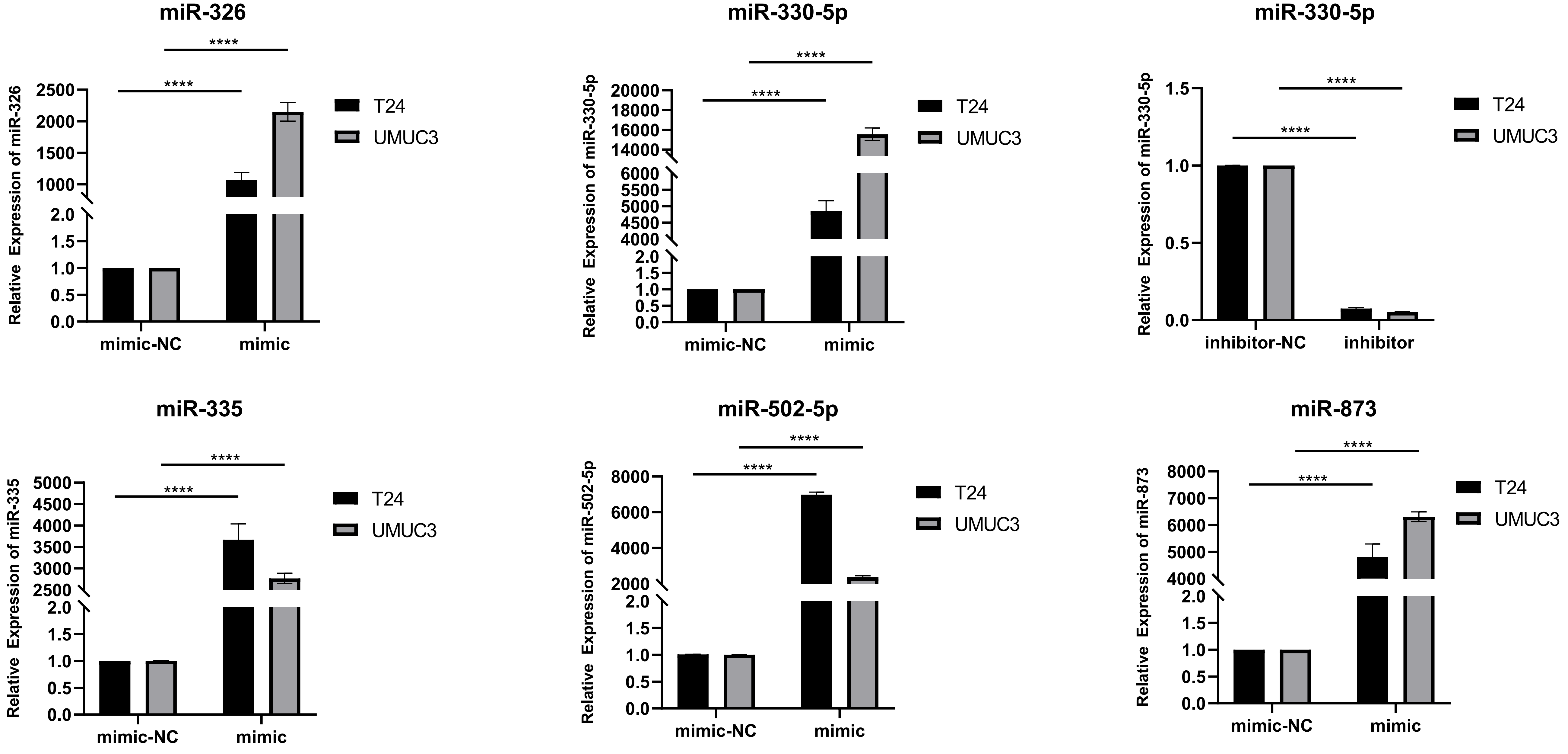
miR-326
Relative Expression of miR-326
mimic-NC
mimic
T24
UMUC3
****
miR-330-5p
Relative Expression of miR-330-5p
mimic-NC
mimic
miR-330-5p
inhibitor-NC
inhibitor
miR-335
Relative Expression of miR-335
miR-502-5p
Relative Expression of miR-502-5p
miR-873
Relative Expression of miR-873

B

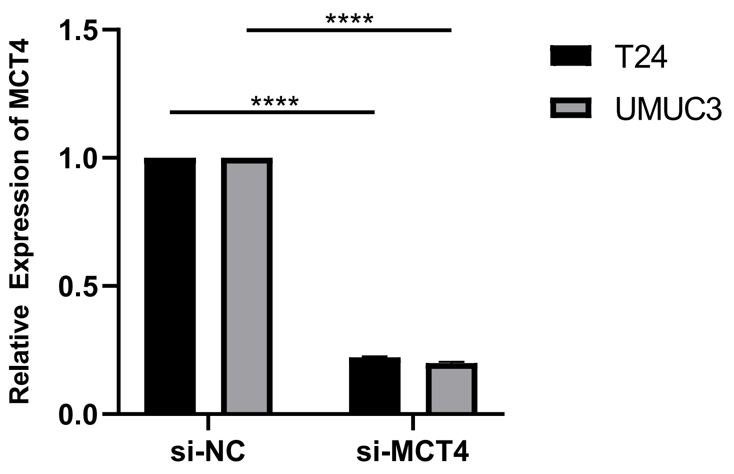
Relative Expression of MCT4
si-NC
si-MCT4
T24
UMUC3
****

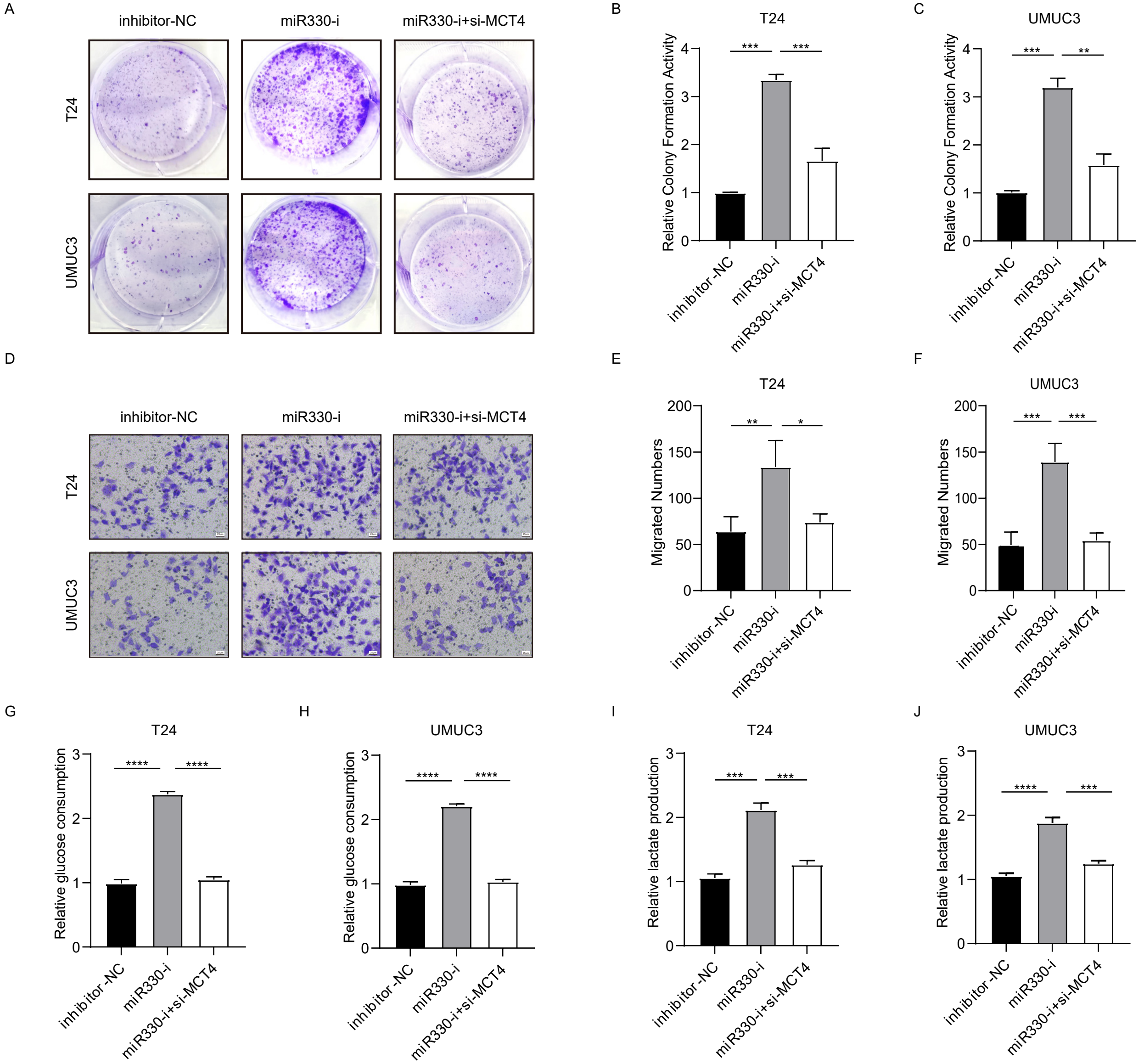

A
inhibitor-NC
miR330-i
miR330-i+si-MCT4
T24
UMUC3
B
T24
Relative Colony Formation Activity
0
1
2
3
4
***
***
inhibitor-NC
miR330-i
miR330-i+si-MCT4
C
UMUC3
Relative Colony Formation Activity
0
1
2
3
4
***
**
inhibitor-NC
miR330-i
miR330-i+si-MCT4
D
inhibitor-NC
miR330-i
miR330-i+si-MCT4
T24
UMUC3
E
T24
Migrated Numbers
0
50
100
150
200
**
*
inhibitor-NC
miR330-i
miR330-i+si-MCT4
F
UMUC3
Migrated Numbers
0
50
100
150
200
***
***
inhibitor-NC
miR330-i
miR330-i+si-MCT4
G
T24
Relative glucose consumption
0
1
2
3
****
****
inhibitor-NC
miR330-i
miR330-i+si-MCT4
H
UMUC3
Relative glucose consumption
0
1
2
3
****
****
inhibitor-NC
miR330-i
miR330-i+si-MCT4
I
T24
Relative lactate production
0
1
2
3
***
***
inhibitor-NC
miR330-i
miR330-i+si-MCT4
J
UMUC3
Relative lactate production
0
1
2
3
****
***
inhibitor-NC
miR330-i
miR330-i+si-MCT4

circ235

vector

circ235+sh-MCT4

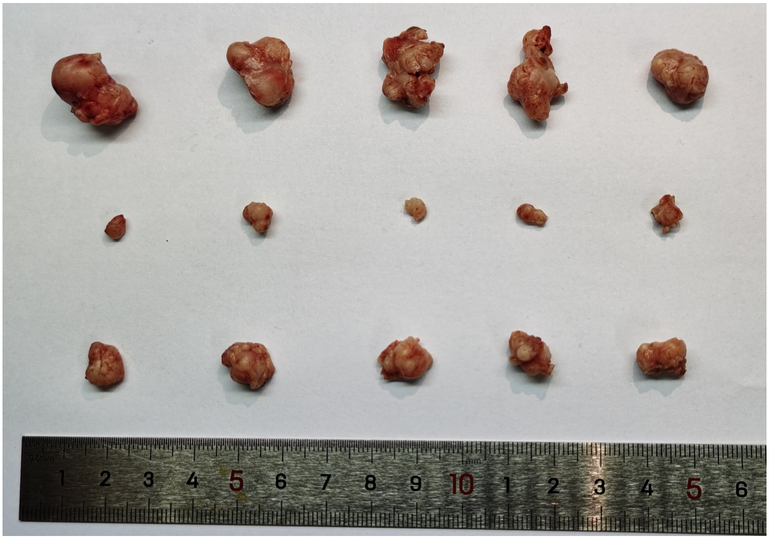

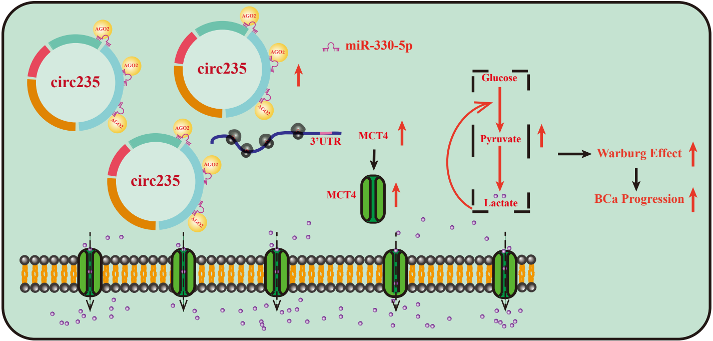
miR-330-5p
circ235
AGO2
3'UTR
MCT4
MCT4
Glucose
Pyruvate
Lactate
Warburg Effect
BCa Progression
