# Supplementary figures and images for: Circ_0000235 targets MCT4 to promote glycolysis and progression of bladder cancer by sponging miR-330-5p
Source: Cell Death Discov. 2023 Aug 2;9:283. doi: 10.1038/s41420-023-01582-z (PMC10397263; doi:10.1038/s41420-023-01582-z)

Fig.5 H-I

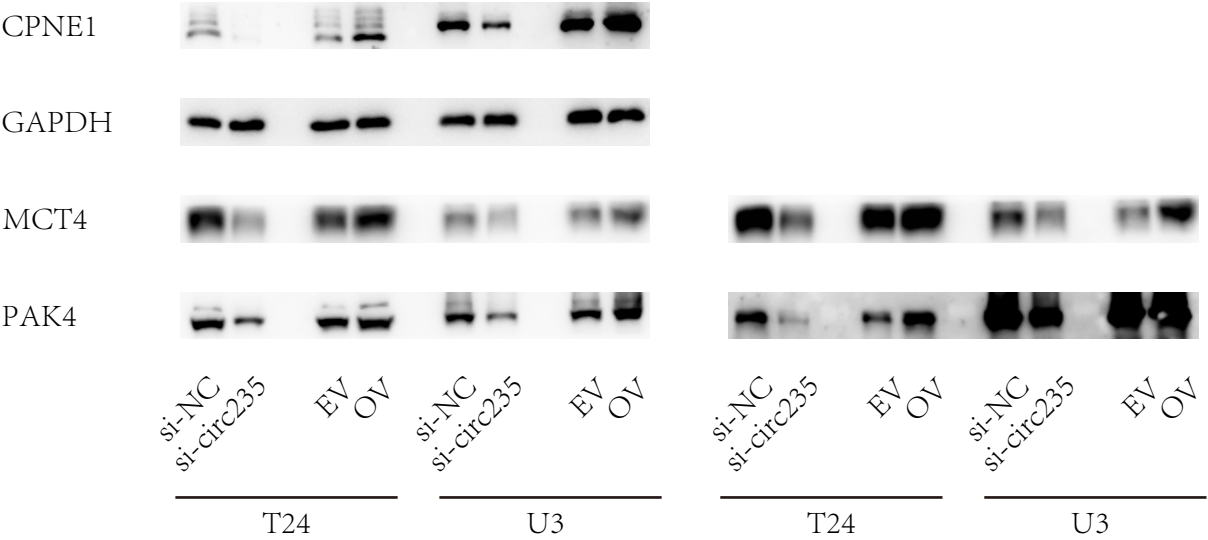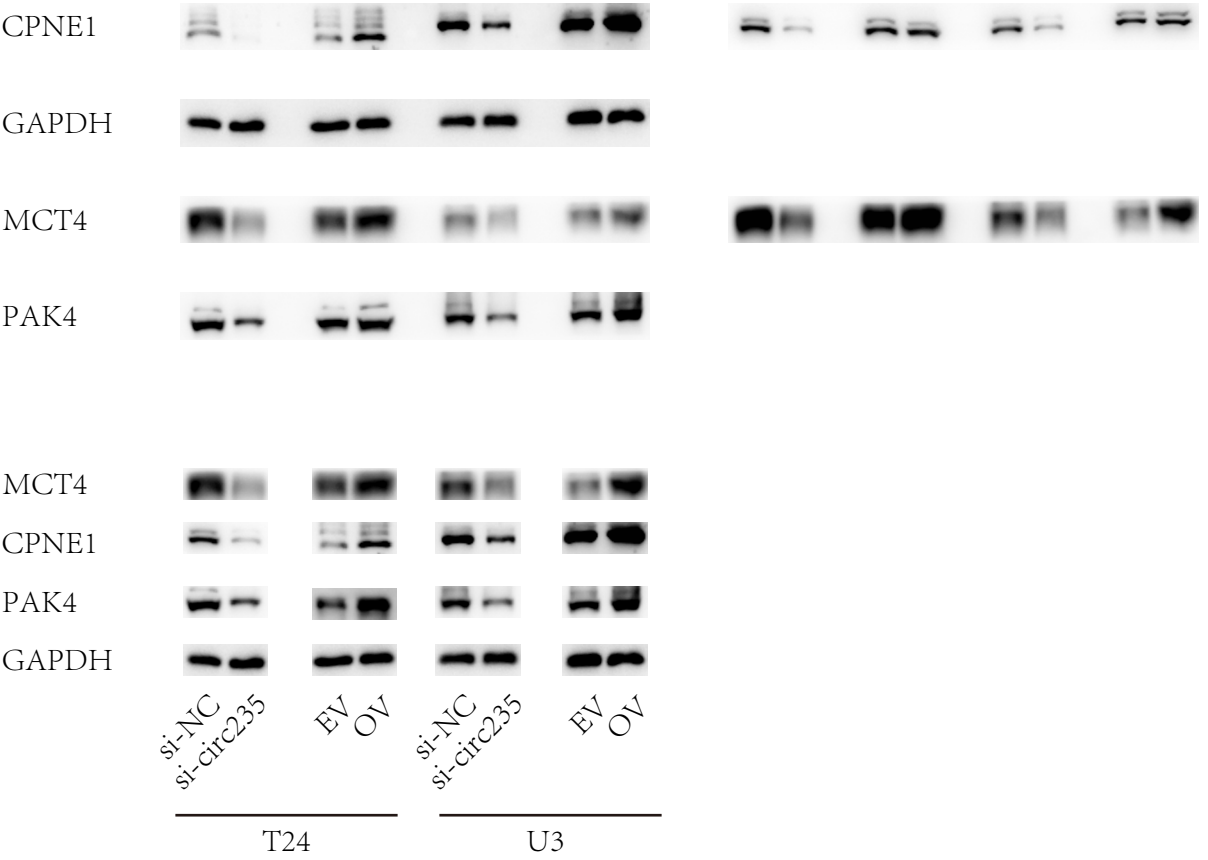

Fig.6 M-N

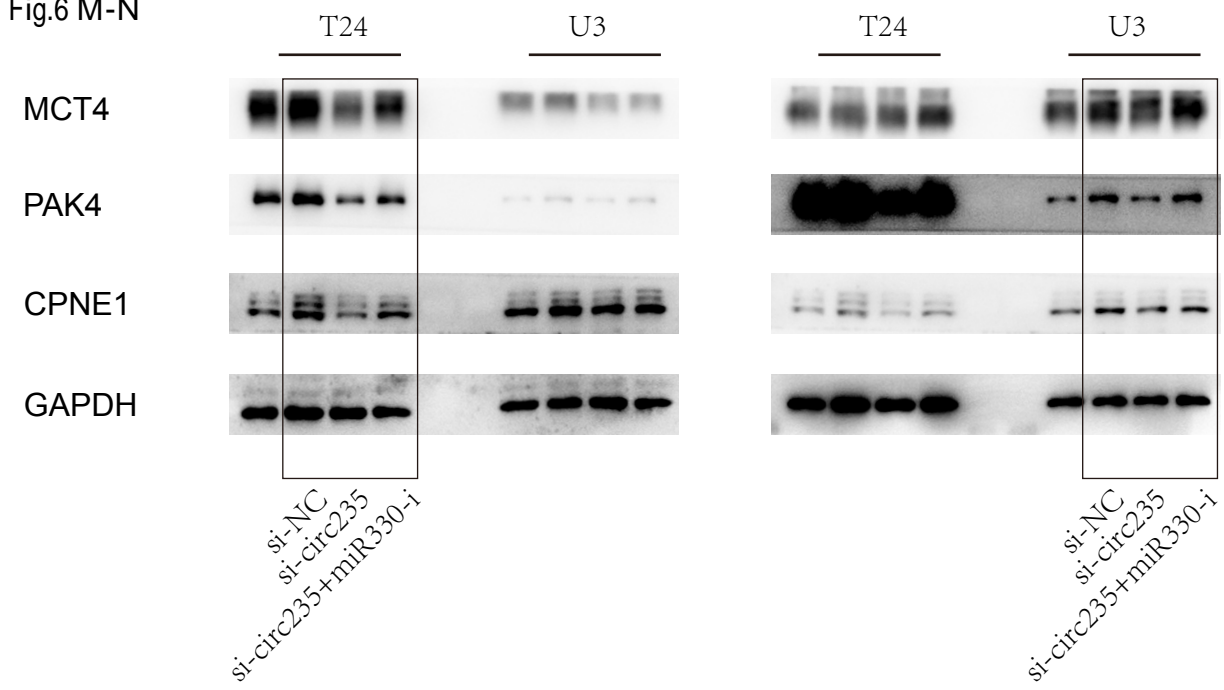

Fig.5M

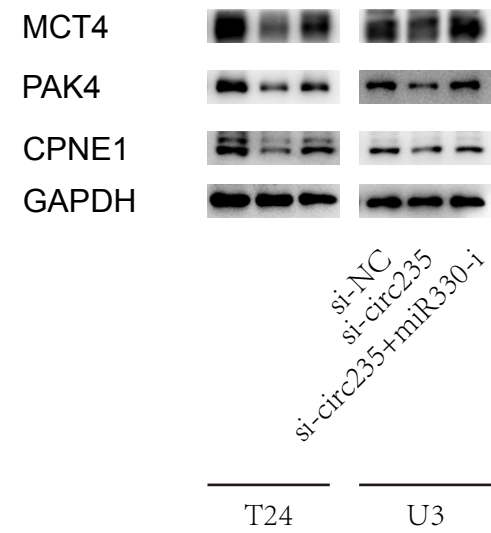

Fig.5N

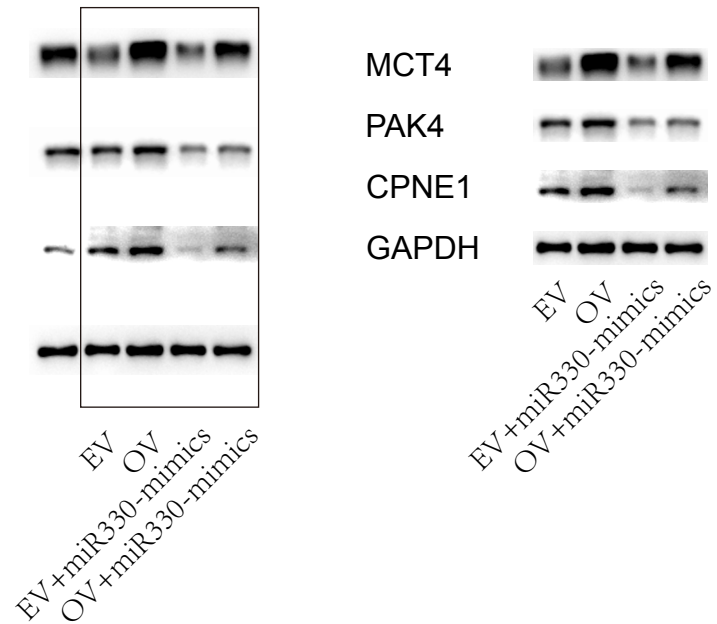

Supplement: Supplementary file 2 — Original Data File [file 41420_2023_1582_MOESM2_ESM.pdf]
