# Supplementary material for: Circ_0000235 targets MCT4 to promote glycolysis and progression of bladder cancer by sponging miR-330-5p
Source: Cell Death Discov. 2023 Aug 2;9:283. doi: 10.1038/s41420-023-01582-z (PMC10397263; doi:10.1038/s41420-023-01582-z)
Supplement: Supplementary file 3 — Supplementary Figure Legends [file 41420_2023_1582_MOESM3_ESM.docx]

**Figure S1.** **Validation of the efficiency of miRNA mimics, miR-330-5p inhibitor, and si-MCT4 A** miRNA relative expression was quantified using qRT-PCR in Bca cells transfected with miRNA mimics or miR-330-5p inhibitor and normalized using *U6*. **B** MCT4 relative expression was quantified using qRT-PCR in Bca cells transfected with si-MCT4 and normalized using *β-actin*.

**Figure S2.** **miR-330-5p regulates the migration, proliferation, and aerobic glycolysis of Bca cells via MCT4. A**, **B**, **C** Exemplary photographs (**A**) and statistical diagrams (**B**, **C**) of colony formation assays were implemented using three sets of T24 or UMUC3 cells co-transfected with miR-330 inhibitor or the control and with or without si-*MCT4* (inhibitor-NC, miR-330 inhibitor, and miR-330 inhibitor+si-*MCT4*). si-*MCT4* partially rescued the proliferative capacity of transfected Bca cells with miR-330 inhibitor. **D**, **E**, **F** Exemplary photographs (**D**) and statistical diagrams (**E**, **F**) of transwell tests were used to evaluate migratory capabilities of the three sets of transfected T24 and UMUC3 cells with miR-330 inhibitor or the control and transfected with or without si-*MCT4* (inhibitor-NC, miR-330 inhibitor, and miR-330 inhibitor+si-*MCT4*). si-*MCT4* still partially rescuing the migration of Bca cells transfected with miR-330 inhibitor. **G**-**J** Relative glucose consumption (**G**, **H**) and relative lactate production (**I**, **J**) in three sets of Bca cells (inhibitor-NC, miR-330 inhibitor, and miR-330 inhibitor+si-*MCT4*) were calculated using the glucose or lactate assay kit.

**Figure S3.** Three groups of T24 cells were injected hypodermically into mice, and typical photographs of tumors were obtained after 19 days.

**Figure S4. Mechanisms underlying circ235-mediated Bca progression.** Circ235 inhibits the binding of *miR-330-5p* to the 3'UTR of the downstream target gene, *MCT4*, through sponge adsorption of *miR-330-5p*, thereby stimulating *MCT4* expression. Upregulation of *MCT4* expression promotes lactate efflux, which subsequently accelerates glycolysis in tumor cells through a positive feedback pathway to maintain intracellular metabolic homeostasis. These results further enhance the Warburg effect and reprogram cellular metabolic pattern, which ultimately facilitates Bca progression.
